# Diverse genetic error modes constrain large-scale bio-based production

**DOI:** 10.1038/s41467-018-03232-w

**Published:** 2018-02-20

**Authors:** Peter Rugbjerg, Nils Myling-Petersen, Andreas Porse, Kira Sarup-Lytzen, Morten O. A. Sommer

**Affiliations:** The Novo Nordisk Foundation Center for Biosustainability, Technical University of Denmark, Building 220, DK-2800 Kongens Lyngby, Denmark

## Abstract

A transition toward sustainable bio-based chemical production is important for green growth. However, productivity and yield frequently decrease as large-scale microbial fermentation progresses, commonly ascribed to phenotypic variation. Yet, given the high metabolic burden and toxicities, evolutionary processes may also constrain bio-based production. We experimentally simulate large-scale fermentation with mevalonic acid-producing *Escherichia coli*. By tracking growth rate and production, we uncover how populations fully sacrifice production to gain fitness within 70 generations. Using ultra-deep (>1000×) time-lapse sequencing of the pathway populations, we identify multiple recurring intra-pathway genetic error modes. This genetic heterogeneity is only detected using deep-sequencing and new population-level bioinformatics, suggesting that the problem is underestimated. A quantitative model explains the population dynamics based on enrichment of spontaneous mutant cells. We validate our model by tuning production load and escape rate of the production host and apply multiple orthogonal strategies for postponing genetically driven production declines.

## Abstract (web summary)

The declining performance of scale-up bioreactor cultures is commonly attributed to phenotypic and physical heterogeneities. Here, the authors reveal multiple recurring intra-pathway error modes that limit engineered *E*. *coli* mevalonic acid production over time- and industrial-scale fermentations.

## Introduction

Bio-based production of chemicals and fuels is important to develop a more sustainable society. However, it remains difficult to scale-up many processes that rely on engineered organisms to produce industrially relevant quantities of bio-compounds, which frequently require 100 m^3^ fermentation volumes. Indeed, a lack of robustness of synthetic production strains is considered a main challenge for implementing large-scale bioprocesses^1,2^. Furthermore, despite advantages such as higher volumetric productivity, the industrial implementation of continuous fermentation is often limited by appearance of non-producer cells^3–5^. Indeed, declining productivity constrains the economic feasibility of most fermentation reactions to shorter fed-batch operations^6^, ultimately limiting our societal transition toward bio-based chemical and fuel production.

Poor performance of bio-based processes is speculated to arise from phenotypic cell-to-cell variation rather than single-nucleotide polymorphisms (SNPs)^7,8^. Suboptimal physical reactor conditions such as limited aeration and stochastic gene expression are thought to underlie population heterogeneities^9–11^. As such, subpopulations have been observed to temporarily cease production, then resume production at an unpredictable time^12^. In addition, the high-level cellular biosynthetic activity required for economically viable bioprocesses might reduce the fitness of producer cells enough to select for non-producing mutant cells during industrially relevant timescales. Such genetic heterogeneity would be more detrimental than temporal phenotypic variations, as genetic heterogeneity results in the irreversible loss of production from a subpopulation in the fermentation tank.

The fitness cost of biosynthesis is pathway-specific and arises from metabolic loads such as enzyme synthesis, DNA synthesis, protein misfolding, and drains on endogenous metabolites (required for glycolysis and redox power), but it can also result from the accumulation of toxic intermediates and by-products^13–18^. We employ the term “production load” to the sum of these effects, which present a selective disadvantage for productive cells in direct competition with non-productive cells. The fitness of a production organism can be improved in a variety of ways, including rational engineering^19^, adaptive laboratory evolution^20,21^, functional metagenomics^22^, and fermentation optimization^23,24^. Despite recent progress, production organisms still retain a fitness cost that cannot be eliminated that is directly linked to the burden of non-natural biosynthetic productivity. Accordingly, production cells may be selected against in competition with more fit non-producing cells. However, the extent to which such evolutionary processes limit fermentation output remains unclear and depends on eventual population size, production load, and the number of cell divisions required to reach industrial fermentation scales.

Generating the fermentation population inside an industrially sized 200 m^3^ fed-batch bioreactor involves a gradual scale-up from a master cell bank aliquot and requires approximately 60–80 cell generations to reach population sizes of approximately 10^20^  cells. Such timescales and population sizes could allow for both the generation and selection of non-producing organisms and might allow these organisms to reach substantial densities in the final fermentation population.

One mechanism that led to non-producing cells in early, engineered bioprocesses is the loss of plasmids that encode components of the biosynthetic pathway. Strategies have been developed to limit the loss of plasmid-borne pathway cassettes, including punishing mis-segregation using plasmid-encoded selection genes, toxin-antitoxin systems, and chromosomal integration of the pathway genes^25–27^. However, maintenance of the biosynthetic pathway cassette does not preclude the accumulation of genetic errors targeting pathway genes or central metabolic host genes in *trans*, which leads to a loss of biosynthetic activity and potentially improved fitness. Indeed, limiting the mutation rate in *Escherichia coli* by deleting error-prone DNA polymerases and chromosomal insertion sequences (ISs) has led to higher end-point l-threonine productivity and overexpressed recombinant protein titer^28,29^. Such reports suggest that genetic heterogeneity resulting from processes other than gene loss might play a key role in limiting fermentation productivity. However, the actual mechanism and population-level dynamics of such genetically driven production disruption remains poorly understood, preventing the establishment of a framework for explaining and addressing such production failure modes.

In this study, we investigate the phenotypic and genotypic dynamics of *E*. *coli* strains engineered to produce mevalonic acid over timescales relevant to industrial-scale fermentations. Mevalonic acid is a precursor to the important secondary metabolite class of isoprenoids, acting as a chemical building block for colorants, medicines, flavors, fuels, and fragrances^30^. Using ultra-deep, time-lapse sequencing of the fermentation populations, we resolve diverse, previously difficult-to-decipher, and non-canonical IS transposition events that limit production.

## Results

### Stability of the mevalonic acid-producing phenotype

**Fig. 1:**
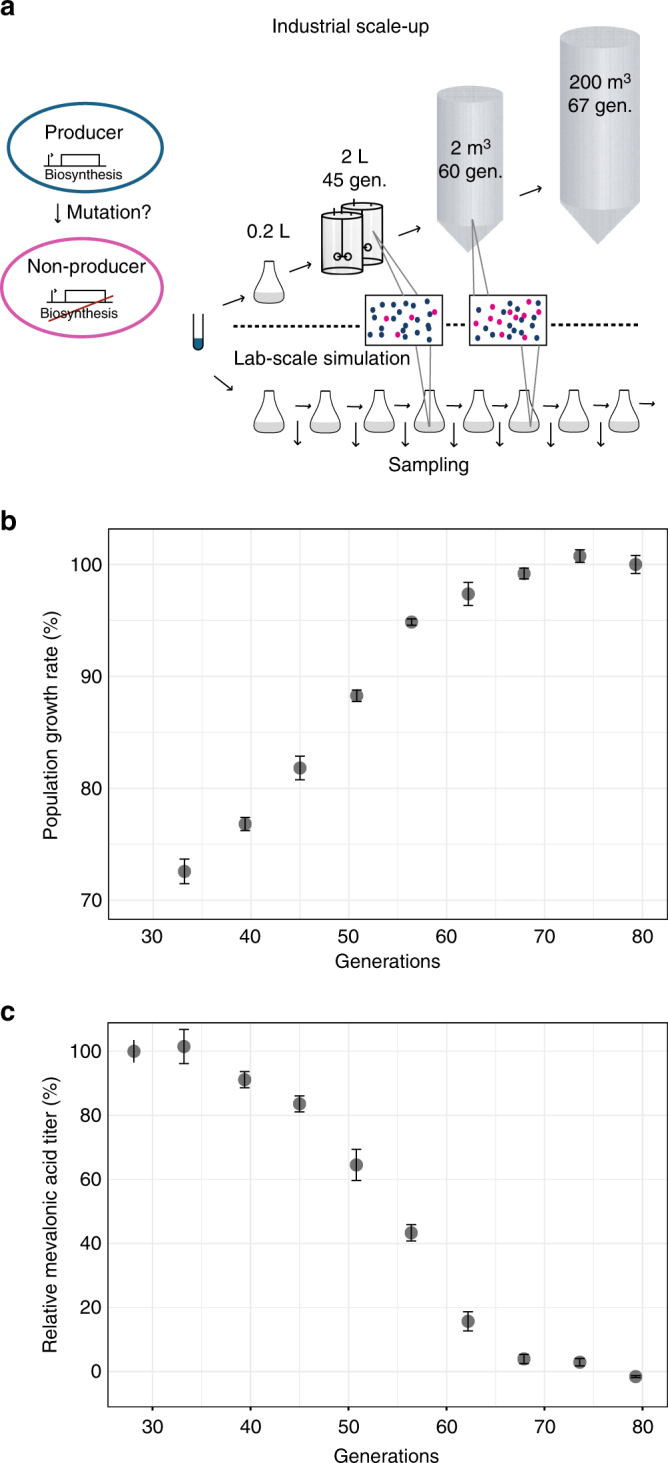
Stability of the mevalonic acid-producing phenotype. **a** Large-scale industrial production of mevalonic acid was simulated through serial transfer of five parallel mevalonic acid-producing populations. The length of the fermentation simulation was chosen to mimic the generation number of a fermentation population in a 200 m^3^ fermentation tank. Production populations were sampled every 8 h for subsequent phenotypic and genotypic analysis (Supplementary Table 2). **b** Population-level average local growth rates were determined for parallel populations over the course of the experiment (Methods). The means are shown relative to the last time point (absolute value 0.84/h). A transition of the mean population growth rate is observed after 35 generations, in which the population growth rate increases to a stable phenotypic state after 70 generations, alleviating the measured production load (Supplementary Fig. 1). **c** Mevalonic acid titers during the simulated fermentations. The means are shown relative to the earliest time point (Supplementary Table 3) and were calculated from five parallel lineages of the *E*. *coli* TOP10 mevalonic acid-producing clone. Error bars denote s.e.m. (*n* = 5)

We wanted to study the phenotypic dynamics of mevalonic acid-producing *E*. *coli* over industrially relevant timescales. Inoculation of large fermenters typically involves gradual scale-up from an aliquot of a master cell bank by serial growth in vessels of increasing volume^4^. During these cultivations, the original clone, giving rise to the master cell bank aliquots, proliferates through >60 cell generations (Supplementary Table 1). To experimentally simulate this growth process, we serially transferred production strain lineages every 8 h for a total of nine times, corresponding to approximately 80 cell divisions (generations; Fig. 1a). Specifically, we cultured five parallel lineages of an *E*. *coli* TOP10 clone harboring an induced mevalonic acid pathway plasmid (pMevT) maintained under constant antibiotic selection to prevent plasmid loss (Methods). To analyze phenotypic and genetic population dynamics, we sampled and freeze-stocked the growing populations every 8 h.

Engineered biosynthesis of mevalonic acid proceeds from acetyl-CoA units through condensation to acetoacetyl-CoA (by AtoB) and 3-hydroxy-3-methyl-glutaryl-CoA (HMG-CoA; by ERG13) prior to reduction with NADPH to form mevalonic acid (by tHMGR)^30–32^. Growth impairment from mevalonic acid production in *E*. *coli* arises largely from the HMG-CoA intermediate, which interferes with central fatty acid metabolism and the cell membrane^19^. As a result of high-level mevalonic acid production, our production strain had a 30% production load, measured relative to a non-producing control strain harboring a pathway-excised plasmid (Supplementary Fig. 1).

To assess the dynamics of the population fitness during the experiment, we evaluated population growth rates (Methods). The average population growth rate gradually increased as a function of generation number, following a sigmoidal pattern that stabilized at a new level after 60–75 generations (Fig. 1b). The population growth rate at the beginning of the experiment was 28% below the final population growth rate, highlighting a considerable change in fitness of the simulated fermentation populations (Fig. 1b). This factor combines all fitness changes over the simulated fermentations, e.g., also possible remaining loads of a disrupted pathway. Notably still, the difference was similar to the measured production load (30%).

Next, we determined the mevalonic acid titer of each sampled population throughout the simulated fermentation (Fig. 1c). Starting from generation 34, product titers began a decline by several percent per generation before leveling off at undetectable concentrations around generation 70. The onset of the decline in mevalonic acid production coincided with the increase in population growth rate and followed an inversely proportional pattern to the increased growth rate.

Population-level growth rates were negatively highly correlated with production titers following an exponential decline (*R*^2^ = 0.99; Supplementary Fig. 2). This correlation demonstrates how mevalonic acid production was reduced as more fit non-producers took over the fermentation population.

### Modeling and measuring production decline at scale

**Fig. 2:**
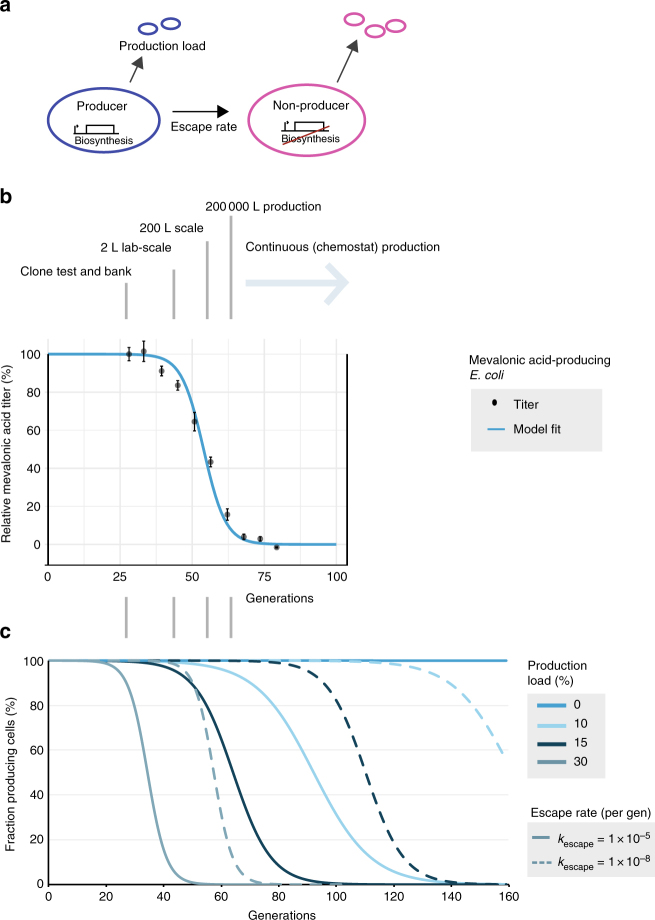
Mathematical modeling is consistent with the observed mevalonic acid production titer. **a** Producer cells mutate from the production state at a specific escape rate, thereby alleviating the production load (fitness cost of production; Supplementary Note 1). **b** The best fit of the mathematical model (Supplementary Table 5) to the observed mevalonic acid titer throughout laboratory-simulated mevalonic acid fermentations (relative to earliest time point, Supplementary Table 3). Error bars indicate the s.e.m. (*n* = 5). For reference, possible reactor sizes corresponding to particular generation numbers are shown (Supplementary Table 1). **c** Modeled fractions of producer cells remaining in the population, in which producer cells irreversibly mutate to non-producers at various escape rates. The magnitude of the production load drives the rate by which spontaneously formed non-producers will enrich the population

Antibiotic resistance and plasmid loss dynamics have previously been studied using population dynamical models^33–36^, but related analysis has not expanded to gene instabilities of metabolically engineered production organisms. To elucidate evolutionary factors of biotechnological production decline, we developed a simple, two-state deterministic model for the population structure of engineered production strains during fermentation (Fig. 2a, Supplementary Note 1). In our model, a fermentation population contains producing and non-producing cells with different growth rates resulting from the production load. The escape rate describes the transition of producing cells to non-producing cells and represents the combined action of all disruptive mutations that abolish the production load (Fig. 2a). This escape rate depends on numerous factors, including host mutation rate, the size of the genetic targets that abolish production when mutated, and the susceptibility of the genetic targets to recombination or other deleterious genetic events. Owing to production load, the producer cells will be gradually outcompeted by non-producers. The magnitude of the production load determines the rate by which spontaneously formed non-producing cells will enrich in the population. This model offers a simplified description of the population dynamics during fermentation and can be represented with two coupled ordinary differential equations (Supplementary Note 1). Solving these equations yields the respective growth functions of producing and non-producing cells over time, assuming a single producing cell as the starting point, constant escape rate, production load, and no nutrient limitation (Supplementary Note 1). To incorporate effects of likely discrete escape events, we also generated a stochastic version of our model (Supplementary Note 4). However, for large populations (>1000 cells), a deterministic model captures an average of the population dynamics and is computationally more efficient due to the existence of an exact analytical solution (Supplementary Table 4)^37,38^.

To assess the applicability of our model, we fitted it to the experimentally determined production stability (relative product titers) by nonlinear regression to predict the escape rate and production load (Fig. 2b). Using the experimentally determined production load (30%, Supplementary Fig. 1), the model estimated an effective escape rate of 2.5 × 10^–8^/generation (95% confidence interval (CI_95%_): ±1.2 × 10^–8^; Supplementary Table 5). Such good fit of the production stability data is consistent with our assumption of a genetic basis for production decline.

Our model describes how the fraction of producing cells in the fermentation population will decline sigmoidally over time when a production load is involved in bio-based production (Fig. 2c). Notably, the initial decline determined by the escape rate is low (Fig. 2c) and difficult to detect phenotypically. The production load mainly determines the half-life steepness of this transition, whereas the escape rate largely shifts the timing of the transition. To explore this concept, we calculated the fraction of producer cells over time in specific cases, with production loads of 0–30% and escape rates of 10^−5^–10^−8^/generation (Fig. 2c). We found that slight changes to either parameter have dramatic consequences for the maintenance of producing cells in a fermentation population. These model predictions describe how significant improvements in fermentation end-performance can result from reductions in production load or escape rate.

### Production decline originates within pathway genes

Many genome-encoded cell functions are necessary for maintaining biosynthetic production, and any disruption offers an evolutionary trajectory for an engineered strain to regain fitness at the expense of production. In a cell factory strain, proteome and genome adaptations might limit metabolic productivity through changes to specific or global transcription factors, protein folding control, or precursor fluxes. We therefore wanted to test whether the production plasmid from the evolved populations still conferred mevalonic acid production to a non-evolved host. Plasmid populations were extracted from the five end points and re-introduced into fresh *E*. *coli* TOP10 strains. The transformed cultures did not show any detectable mevalonic acid production, demonstrating that the mevalonic acid pathway had been disrupted to incapacitate its biosynthetic potential. Additionally, we found no SNPs in the genomes of nine randomly selected colonies from the end-point populations relative to the ancestral strain (Supplementary Table 6).

### Ultra-deep-sequencing reveals pathway disruption dynamics

To investigate the genetic basis of production failure at the population level, we ultra-deep-sequenced the heterologous mevalonic acid biosynthetic pathway from three lineages at five sampling points during the experimentally simulated fermentation, and all five at the generation 70 end point (paired-end 2 × 150 bp Illumina sequencing at average depth of 7200×, Methods).

**Fig. 3:**
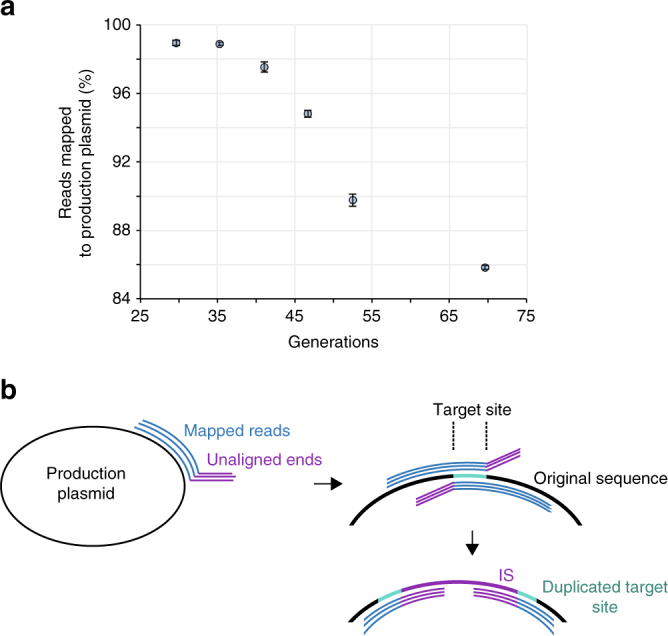
Inferring invasion of deep-sequenced fermentation cell populations by foreign genetic material. **a** Percentage of total reads mapped to the production plasmid sequence declined over the course of the fermentation (cell generations; error bars depict s.e.m., *n* = 3, except generation 70: *n* = 5). **b** Analysis of mapped reads indicated insertion sequence (IS) transposition owing to the presence of crossed-over broken read mappings representing IS insertion and the duplication of target sites, also evident from elevated target site sequencing coverage (Supplementary Fig. 4)

Prior studies of cell heterogeneity applied SNP analyses to cultured production strains, yet these studies found no evidence of genetic variance^7^. We similarly found a lack of SNPs above 1% frequencies in our mevalonic acid pathway end-point populations (Supplementary Fig. 3). However, we observed that a declining share of the reads mapped to the production plasmid sequence (Fig. 3a), indicating structural rearrangements of the biosynthetic pathway or other critical parts. This observation prompted us to develop a bioinformatics approach to analyze genetic heterogeneity focusing on structural rearrangements and insertions.

**Fig. 4:**
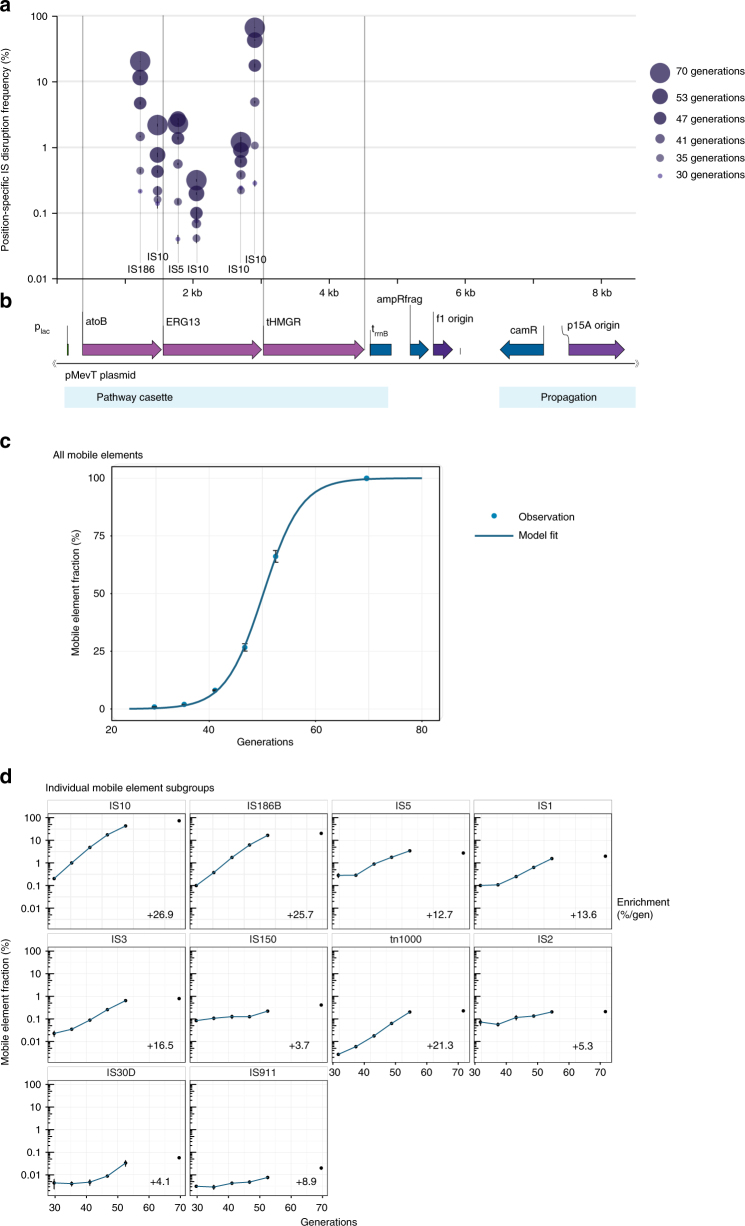
Genetic pathway stability of a mevalonic acid-producing *E*. *coli* TOP10 clone in parallel lineages. **a** Time-lapse high-depth sequencing revealed rising frequencies (population fractions) of mobile element insertions. **b** The production plasmid pMevT, which encodes the genes *atoB*, *ERG13*, and *tHMGR* in the mevalonic acid pathway. **c** Total enrichment of mobile elements in plasmid populations over the experimentally simulated fermentation period along with the model fit. **d** Individual enrichment of host mobile elements in production plasmid populations over the simulated fermentation, and their percent-wise enrichment per generation in the exponential range (generations 30–53; regression statistics in Supplementary Table 7). For all the graphs, the error bars depict s.e.m. (*n* = 3, except at generation 70: *n* = 5)

Notably, we observed that reads not mapping to our production pathway frequently exhibited a near-perfect partial alignment to our production pathway. We termed such reads broken reads and focused our analysis on these reads (Methods). The consensus sequence of several of the unaligned broken read ends showed perfect identity to the termini of ISs and tn1000 (also known as gamma-delta), which are mobile elements resident in the *E*. *coli* TOP10 genome. Accordingly, we speculated that these broken reads result from integration of ISs within the production pathway. Notably, the gap between the right and left breakpoints usually exhibited signature lengths of 3–12 bp, suggesting possible IS target sites (Fig. 3b). At several high-frequency IS target sites, a clear rise in insertion site coverage was observed, likely resulting from duplication of the target insertion region (Supplementary Fig. 4)^39^. To quantify disruption dynamics, we tracked the fraction of position-specific coverage relative to corresponding coverage of non-disrupted reference sequences (Fig. 4a; Methods). We generally detected the position-specific presence of such disruptions in the pathway populations at frequencies down to 0.04% (three reads).

We found that during the experimentally simulated fermentation, six specific positions in *atoB* and *ERG13* of the metabolic pathway were disrupted by IS10, IS186, and IS5 insertions, jointly constituting >91% at the end points, generation 70 (Fig. 4a, b). While the *atoB* and *ERG13* pathway genes became increasingly disrupted during the experiment, the final pathway gene *tHMGR* remained free of disruptions (Fig. 4a). This striking degree of preservation of the *tHMGR* gene is probably due to the cytotoxicity of HMG-CoA^19^, the substrate of tHMGR. Spontaneous mobile element disruptions of *tHMGR* likely became toxic in cells with active *atoB* and *ERG13*, as these cells would accumulate cytotoxic HMG-CoA concentrations. Because several *atoB* disruptions were also enriched despite the presence of a chromosomal copy, it is very likely that enriched insertions within *atoB* also abolish *ERG13* activity by means of IS-mediated transcriptional termination^40^ owing to the operon structure of the mevalonic acid biosynthetic pathway.

Given that complete production loss was observed in the populations, we speculated that other mobile elements could explain the remaining 9% fraction. As a strategy to fully resolve the population reads, we mapped all reads to the 24 unique mobile element subgroups in the *E*. *coli* DH10B genome^41^, i.e., not detecting for loci-specific dynamics (Methods). We found that joint mobile element coverage relative to the original pMevT approached 99.9% (s.e.m. = 1.1%) at generation 70 (Fig. 4c). A spectrum of 10 host mobile element subgroups each transposed to a frequency above 0.01% in the end-point populations (Fig. 4d).

Using ultra-deep-sequencing data to infer population structure is more direct than relative production titers because of prior knowledge of the initial, pure starting point and no requirement for sample re-cultivation. By fitting the time-resolved total mobile element fractions to our production stability model, we improved the confidence of the prediction and estimated an escape rate of 8.7 × 10^−8^/generation (CI_95%_: ±0.2 × 10^−8^; Supplementary Table 5). We therefore fitted our data without a pre-determined production load to see how well the model could estimate both parameters freely. From sequencing-based stability data alone, the model very confidently predicted an alleviated production load of 28.1% (CI_95%_: ±0.1%) and a revised escape rate of 2.1 × 10^–7^/generation (CI_95%_: ± 0.1 × 10^–7^; Supplementary Table 5). The predicted 28.1% production load is notably similar to the experimentally determined 30% production load. An escape rate of 2.1 × 10^–7^/generation corresponds well to previously observed mobile element transposition rates into the selectable *cycA* gene in *E*. *coli* DH10B^41^. The escape rate of our simple model assumes a complete cellular transition from producing to non-producing behavior upon escape. Within each cell, escape begins with a single plasmid mutation, which upon cell divisions is increasingly selected toward a pure non-producing plasmid population (at ca. 15 copies for p15A-origin plasmids^42^), potentially giving rise to an intracellular escape heterogeneity. The process toward intracellular escape fixation is driven by uneven plasmid segregation and increasingly selective advantage with each additional pathway escape. Consequently, the effective production escape rate *k*_escape_ (used in our deterministic and stochastic modeling) captures the average rate of these combined processes and therefore likely underestimates the actual IS insertion rate.

Given the lack of selectable composite elements in ISs such as antibiotic resistance genes, spontaneous insertion rates have traditionally been harder to detect for ISs than for transposons carrying selectable features. Ultra-deep time-lapse sequencing strategies similar to this study should thus be useful to further elucidate the molecular fundamentals of bacterial IS transposition. On a full cell population basis, broad-spectrum enrichment of four unique mobile element subgroups was detected to final frequencies above 1% (Fig. 4d). No apparent correlation between DH10B genomic IS copy number and the enrichment rate was observed (Fig. 4d), as exemplified by subgroups IS2 and IS5, which enriched slowly although present at 12 and 13 genomic copies, respectively. This behavior is in contrast to the 27%/generation enrichment of IS10 and IS186, which are present at only three and four DH10B copies. IS10 and IS186 thus enriched to 93% of the end heterologous mevalonic acid pathway population.

Target site selection of mobile elements is influenced by specific consensus sequences and molecular activities, such as on-going transcription^40^. The consensus IS10 target site was present in *tHMGR*, the p15A origin, and the f1 origin^39^, but insertions were not detected in these loci since disruption of these elements would likely not be advantageous for growth. Instead, IS10 insertions were observed in non-canonical sites within the *atoB* and *ERG13* genes. It is surprising that IS10 disrupts the production load through such non-canonical target consensus (Supplementary Fig. 5), and this observation suggests that the spectrum of some ISs is far wider than previously thought.

Tracking broken read dynamics throughout the experimentally simulated fermentation also allowed us to estimate non-mobile element structural variations that were enriched in the populations, and to compare these variations to those without apparent fitness advantages that merely remained at constant frequencies during the experiment. Structural variations detected in the pathway terminator region were not enriched (Supplementary Fig. 6), and thus likely did not influence production. Such variations might have been observed as artificial noise owing to secondary structure formation during sample preparation. We also searched for signs of direct-repeat homologous recombination, but this was not observed, likely owing to the lack of repetitive sequences larger than 18 bp in the pathway plasmid.

The relatively high estimated production escape rate (2.1 × 10^–7^/generation) is also consistent with the low presence of enriched pathway SNPs (Supplementary Fig. 4), given the basal SNP formation rate of 10^–10^/bp/generation^43^. To assess whether the genetic error modes were host-dependent, we repeated the experiment with similar procedures using a cell bank of a different production strain, *E*. *coli* K-12 strain XL1 (Methods). The end points of five parallel-cultured populations were deep-sequenced to map the error landscape. Comparison of the error modalities in the two different strains revealed both recurrent motifs as well as clone-specific mechanisms (Supplementary Fig. 7). Such strain differences could be due to different chromosomal IS copy numbers. Next, we also wanted to investigate whether the findings of diverse IS insertions were specific to the studied genetic pathway and expression system. We therefore constructed a constitutively expressed mevalonic acid pathway using the different, heterologous genes *mvaS* and *mvaE* of *Lactobacillus casei*^31^ (Supplementary Fig. 8). We experimentally simulated a large-scale fermentation with the pathway plasmid pMVA1 in TOP10 (*h11*). Upon deep-sequencing of the end-point populations (79 generations), we found a diverse error modality again consisting of nine transposed IS subfamily types and low-frequency SNPs (Supplementary Fig. 8, Supplementary Table 8) directly within the expression cassette of the pathway genes. In two lineages (m0 and m2), we found 10 bp deleted from the promoter p_J23100_ (20.2 and 0.2%) and 9 bp deleted the RBS of *atoB* (0.3%, n.d.) without obvious structurally mechanistic clues given in the nucleotide sequence, thus adding such illegitimate, non-homologous recombination to the spectrum of error modes observed. No such short deletions were however detected in the time-lapse sequenced experiment with the pMevT pathway (strain *h2*), indicating that this error mode at this formation rate was plasmid specific.

### Model-guided optimization of production stability

**Fig. 5:**
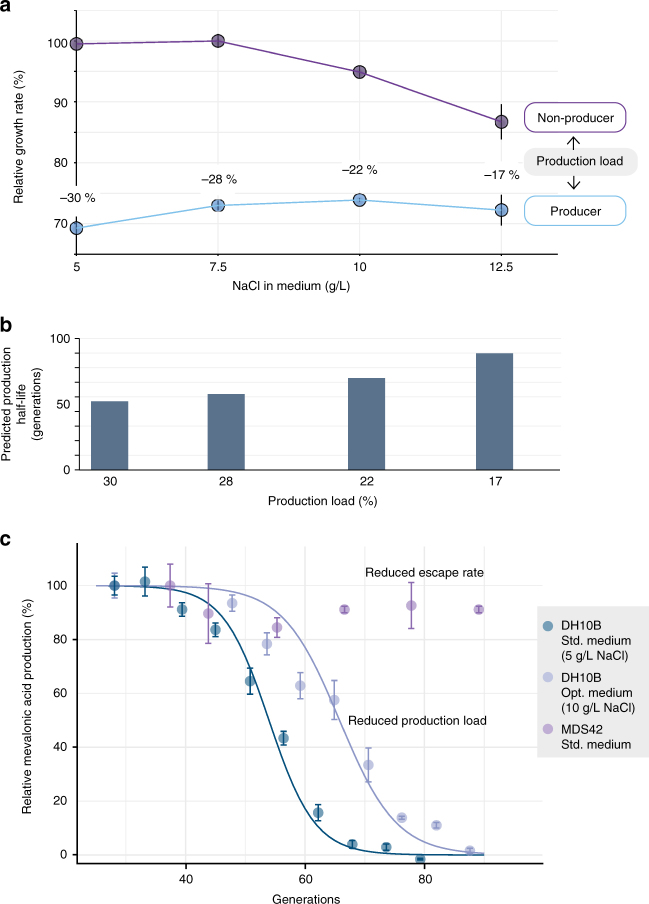
Reducing production decline by optimization of spontaneous escape rate and production load. **a** Limiting the production load of mevalonic acid production by supplementing medium with elevated NaCl concentrations (i.e., minimizing the growth advantage of mutation). This reduction is evident by the growth rate difference of pure producing and non-producing populations. Growth rates depicted relative to fastest growing condition and production load calculated as relative growth rate difference. Error bars depict s.e.m. (*n* = 6). **b** Predicted half-life of a producing cell population with a 2.1 × 10^–7^/generation escape rate and corresponding production loads estimated from modulated NaCl levels in panel **a**. **c** Utilizing a reduced production load and escape rate, respectively, to extend production half-life in experimentally simulated long-term fermentations, evaluated by mevalonic acid production titers (relative to earliest time point; Supplementary Table 3). Results shown for a TOP10 host (*h2m0*) in standard (*n* = 5, blue data points) and in optimized medium (*n* = 4, generation 49: *n* = 3, blue-gray data points) and for an MDS42 host (*h10m0*) with reduced escape rate (*n* = 4, purple data points). Error bars depict s.e.m. Lines represent best fits to population fraction model at a 2.1 × 10^–7^/generation escape rate (Supplementary Table 5). Data for TOP10 host in std. medium also shown in Fig. 2b is included for comparison

As described by the mathematical model, reductions in production load and escape rate represent powerful approaches to improve strain stability. To explore these, we sought to improve production stability by both media and strain engineering to limit the production load and escape rate, respectively. The production load resulting from HMG-CoA cytotoxicity is speculated to result from a destabilized lipid membrane due to inhibitions in early-step lipid biosynthesis^24^, which may be countered by elevated medium osmotic pressure^44^. We therefore quantified the growth rate of mevalonic acid-producing and non-producing populations in a range of NaCl concentrations. At higher osmotic pressure (7.5–12.5 g/L NaCl), we found that the growth rate of pure non-producing cultures was reduced relative to producing cultures, minimizing the original 30% growth inhibition to 18–28% (Fig. 5a).

Our model predicts that a reduction of the production load from 30% to, e.g., 22% would improve the half-life of producing cells from 47 to 63 generations (Fig. 5b). For processes operating in bioreactor scales >50 m^3^, such improved stability could provide a crucial enhancement of the product yield and titer. We thus experimentally simulated another long-term fermentation with the same cell bank (*h2m0*) using one such optimized medium composition (10 g/L NaCl). We measured the mevalonic acid production of the population during the fermentation simulation. The production dynamics showed a clear improvement in stability and a slightly less sharp transition of the population (Fig. 5c), matching the reduced selective advantage of production loss. Indeed, the best model fit to this production stability profile (Supplementary Table 5) estimates a reduced production load at 21% (CI_95%_: ±0.9%). This is very close to the measured production load (Fig. 5a) and corresponds to an extension of the production half-life from 54 to 66 generations. The HMG-CoA accumulation of mevalonic acid production is associated with osmotic and oxidative stress^24^. Cross-protection in osmotic and oxidative stress response of *E*. *coli*^45^ may thus also explain why elevated osmotic pressure in part alleviated the HMG-CoA growth inhibition. The observed, reduced basal mevalonic acid production may also explain the reduced growth inhibition of these cells (Supplementary Table 3).

Reducing the escape rate offers an alternative strategy that is especially favorable when production loads are difficult to minimize. Due to the observed impact of IS-dominated pathway disruptions, a host strain lacking ISs should prevent this escape mechanism. To benchmark the dynamics in such chassis, we transferred the metabolic pathway (pMevT) to a previously generated, genome-reduced, and IS-free *E*. *coli* K-12 strain MDS42^46^. We simulated a long-term fermentation with this host strain using our standard medium and quantified the dynamics of mevalonic acid production (Fig. 5c). The marked improvement in production stability supports the hypothesis that lack of host genomic ISs significantly improves production stability, leading to good preservation of production at the end point of the fermentation simulation (89 generations). However, lower escape rate error modes resulting from SNPs and non-homologous recombination may impact stability on a longer term. Further, the fitness-neutral genome reductions guiding the construction of MDS42^46^ may not necessarily be neutral to metabolic production performance, as indicated by the lower mevalonic acid titer in the MDS42 host (Supplementary Table 3).

### Stabilization by pathway coupled to essential gene

**Fig. 6:**
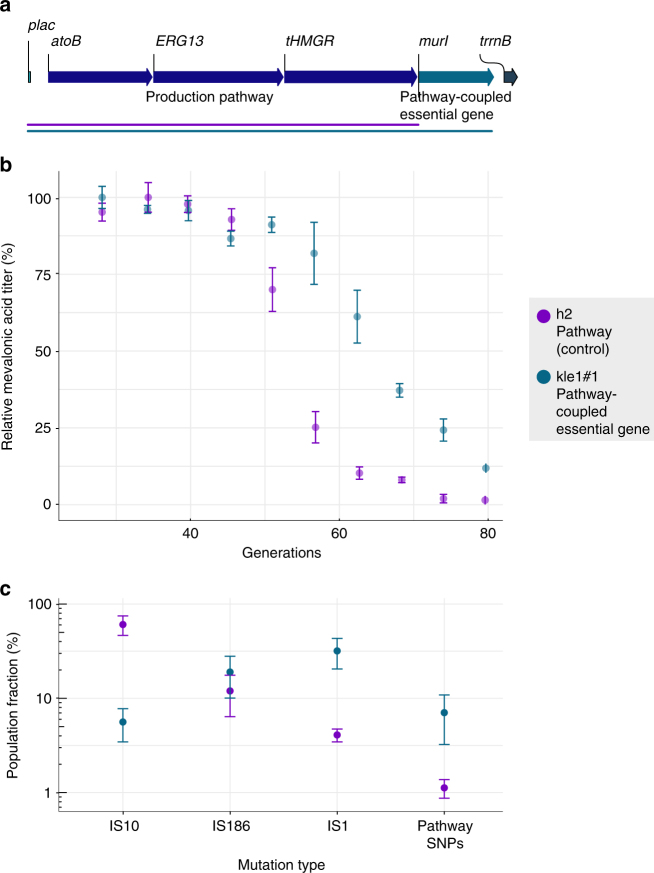
Pathway-coupled essential gene reshapes the long-term production error profile. **a** Genetic pathway design of control (*h2*) and transcriptionally coupled essential pathway gene (*kle1#1*). **b** Mevalonic acid titers (relative to earliest point) (Supplementary Table 3) in experimentally simulated fermentations of control pMevT *h2* and pathway-coupled essential gene strain *kle1#1* (Supplementary Table 9). **c** Fraction (%) in pathway population of the three most abundant pathway-disrupting IS types, compared to summed frequencies of pathway SNPs (Supplementary Table 10). Error bars denote s.e.m. (*n* = 3)

Our pathway sequencing data indicated that the main *atoB* IS disruptions conferred a transcriptional termination of subsequent pathway genes (Fig. 4a). Accordingly, we speculated that evolutionary production decline due to IS disruptions could be reduced by coupling the production pathway transcriptionally to an essential gene. We inserted *murI* into the pathway operon directly following the last pathway gene (Fig. 6a), while directly thereafter deleting the chromosomal copy to avoid recombination and to render the cell dependent on an active pathway operon. *murI* encodes the glutamate racemase, which is not associated with production, but essential for growth^47^.

Compared to essential gene-based stabilization against plasmid loss^48^, such direct pathway stabilization would likely require careful initial titration of essential gene expression, since both elevated and depleted expression levels could reduce cell fitness and thus destabilize the production phenotype. We therefore tested eight different predicted RBS strengths^49^ (Supplementary Table 11). Several of the resulting clones appeared to have too high *murI* translational strength. This might cause a fitness cost to select for transcriptional decline by IS insertion and amplify production instability, as observed in pre-screen serial passing. However, one clone was identified (*kle1#1*) with a predicted very low RBS *murI* strength (Supplementary Table 11), which was slower-growing and appeared more stable in a pre-screen. *kle1#1* was therefore tested in our simulated fermentation setup against the non-coupled pMevT pathway *h2* with wild-type chromosomal *murI*.

As hypothesized, the essential gene-coupled pathway strain maintained mevalonic acid production longer (Fig. 6b) than the non-coupled control, resulting in an extension of the production half-life from 53 to 63 generations. Deep-sequencing of the pathway populations at generation 70 showed that the error profile had changed (Fig. 6c), significantly disfavoring the previously top-enriching IS10 type. This indicates that such IS transposition resulted in an overall negative influence on cell fitness due to a collateral detrimental change in *murI* expression in the *kle1#1* strain. In spite of the extended production stability exhibited by *kle1#1*, we observed an elevated frequency of the previously less active IS1, as well as an elevated abundance of pathway frameshift SNPs (Fig. 6c). The selective enrichment of IS1 may be due to a different influence on neighboring genes, e.g. driven by outwardly-acting promoter activity as previously observed for IS1^40^. Furthermore, SNPs might not be prevented by a transcriptionally coupled essential gene and their rise in frequency relative to IS insertions (Fig. 6c) is therefore expected. Interestingly, the two main pathway frameshift SNPs recurred across the different clone banks of pMevT and pMevT-murI; these insertions/deletions of A and C in *atoB* and *ERG13* targeted mononucleotide repeat stretches and were likely due to DNA polymerase slippage (Supplementary Table 10). Thus, these examples suggest that mononucleotide repeats in critical pathway genetics parts should be tested for and avoided, e.g., as a new, integral part of traditional codon optimization. Overall, the results of the coupled essential gene demonstrate that modifications to the production host to counter specific error modes can lead to the appearance of new error modes, albeit on a longer timescale, resulting in improved pathway stability.

## Discussion

Bio-based production is a central contributor to the transition of our society toward a greener and more sustainable future. However, large-scale bioprocesses are hampered by high yield and productivity requirements, and many new processes cannot be made commercially viable due to declining performance at the scale-up step. Prior work has focused on the phenotypic variance that can contribute to the reduced performance of bio-based processes^50^. While IS elements have been shown to disrupt production phenotypes, the role of evolution and genetic mechanisms in production decline is not well characterized. In this study, we experimentally simulated the timescales and population sizes of large-scale bioprocesses in production of the key biochemical building block, mevalonic acid. We introduced a simple framework that captures population dynamics of engineered production strains. We demonstrated in several different host strains that evolution substantially affects population structure over industrial timescales, with direct ramifications for bio-based process performance. While ultra-deep-sequencing has advanced the understanding of heterogeneities in human disease evolution^51^, so far no studies have investigated the potential for biotechnological evolution at population depth, in part possibly due to difficulties in resolving structural variations by short-read sequencing of populations. We observed that pathway error modes are dominated by a broad spectrum of IS insertions in non-canonical target sites. These remove or alleviate the production load, and the error modes differ between strains and clone banks, but appear rapidly in a population.

We find that two key parameters influence the probability and speed by which evolution can impact cell factory stability: the escape rate, which is the rate by which non-producing mutants are generated in a population; and the production load, which manifests a lower fitness of producing cells in direct competition with non-producers. Based on these parameters, a two-state mathematical model accurately describes the essential population dynamics of experimentally simulated fermentations. By de-convoluting stability into its two principal parameters, the model provides a quantitative framework for evaluating the scale-up process, such as the long-term impact of a loaded pathway enzymatic step. This model describes genetic heterogeneity at the population level and assumes a two-state transition from producer to non-producer cell, which may not be adequate, e.g., for pathways operating with several independently loaded biosynthetic genes. Further, the model assumes growth without nutrient limitations and a constant escape rate and production load. Average experimental estimations appear to approximate the load well in our setup (Supplementary Fig. 1), and may help isolate the production escape rate averages. However, escape rates may be stimulated by different molecular stresses, e.g., in the final phase of bioreactor growth, which are unaccounted for in our simulation. In this study, we have approximated the industrial use of gradually increasing seed train sizes under which most cell divisions occur, by strict passing of cultures in exponential phase throughout the study and shown good fit to a simple model. Thus, integration of dynamic models with time-resolved phenotypic and genotypic data may help guide an investigator to separate load and mutational effects during production strain and process development to more rationally accommodate evolutionary process limitations.

Our results offer a potential explanation of why lab-scale yields and titers might not accurately predict large-scale fermentation performance following a scale-up procedure, despite vector maintenance. The observed pathway disruptions occur within a plasmid population maintained by selection. We find that these modeled dynamics of structural pathway disruption are similar to those of segregational plasmid loss^33^, although they act at different rate scales and are characterized by a diversity of error modes (Fig. 4). As an example, our model predicts that an initially pure producing population, with a production load of 30% and an escape rate of 10^−7^/generation, will shift to 96% non-producing cells over 60 generations, corresponding to a bioreactor of 2 m^3^. Remarkably, the same population at lab-scale (age of 37 generations) might appear high performing with <3% non-producers in the population. Because the majority of product is synthesized when the fermentation population reaches the final density in industrial-scale production, it is crucial to investigate the population genotype at this point and not simply extrapolate phenotypic performance from lab-scale experiments. Time-lapse ultra-deep-sequencing represents a valuable approach for determining error modes, occurrence rates, and their alleviated loads at an early stage. Such ultra-deep-sequencing may also be applied to existing scaled-up fermentations, previously thought to be free of genetic heterogeneity.

Common industrial practice employs production strain clone banks stocked as frozen aliquots. Genetic errors in only a few cells might reside in the starting seed although the population appears healthy for a considerable number of divisions. Seeding fermentations from the same cell bank clone therefore generates a highly recurring stability profile. Cell bank aliquots should therefore be evaluated for rare pre-existing mutations that disrupt production and could be selected for during production scale-up. Deep-sequencing of master cell bank aliquots could be applied to test for this.

Considering that production load improvements by even a few percent can substantially improve stability (Fig. 2c), the specific contributors to production loads must be addressed for each pathway and host cell considering a final large-scale process. For example, reducing the production load from 28 to 23% should extend the production half-life by 10 generations (assuming a constant escape rate of 2.1 × 10^–7^/generation).

Practical strategies will be required to reduce factors of production load, including medium optimization, improved balancing of pathway gene expression, and the cellular export of toxic by- or end products. Poor balancing may favor accumulation of toxic pathway intermediates, which carry particularly high potential as a production load. Because intermediates are intracellular, associated toxicities selectively target the producing cells. In this case, adding genes to degrade toxic by-products or to dynamically redirect pathway flux and the use of specific metabolite- or stress-induced pathway promoters may be advantageous for limiting production load^52–54^. In the rare cases of growth rate-coupled production, semi-continuous processes have been commercialized to improve productivity, such as for R-lactic acid in *Lactobacillus*^55^.

In attempts to improve stability, systems for maintaining metabolic pathways through multiple chromosomal integrations are often used^56,57^ while stabilizing duplications may also result randomly during optimization^58^. Still, integrated pathways remain subject to intra-pathway disruptions by SNPs, mobile genetic elements, and illegitimate recombination, but independence of the individual integration sites means that individual escapes will not be enriched within the intracellular pathway population such as uneven segregation allows in multi-copy plasmid systems. Independently integrated pathway copies may thus provide stabilization in addition to easier antibiotic-free pathway maintenance, which today appears as the major advantage of chromosomal propagation. Yet multiple targeted integrations require significantly longer construction protocols, especially at >50 copies and when limited to IP-free engineering. Future studies should investigate the changes in intra-pathway escape dynamics of such multi-copy, chromosomally integrated production pathways. Based on our results, removal of mobile elements from the genomes of microbial production strains^59^ is a relevant first step for long-term stabilization and the enabling of toxic and burdened pathway expression. Such strategies postpone the onset of significant genetic heterogeneity (Fig. 5c). Mechanistically, escape via homologous recombination points can be avoided, e.g., by synonymous codons^60^. Alternatively, coupling of essential genes to the production pathway operon can also increase production stability (Fig. 6b).

Dynamic fermentation population models might serve as technical tools to predict the necessary reduction in escape rate or production load for a particular bioreactor size. Knowledge of stability dynamics should ensure a more holistic evaluation of strains by taking into account the potential for rapid performance loss. By characterizing and modeling the interplay between spontaneous genetic errors at depth and their selection by metabolic burden and pathway toxicity, we have shown the paths for their synergistic impact on pathway stability. Furthermore, we have demonstrated how engineered reductions in both production load and escape rate can improve stability. We expect that the results, methodologies, and their implications will open new opportunities for metabolic engineers in the quest to develop sustainable and industrially scalable bioprocesses.

## Methods

### Strains

*E*. *coli* K-12 parental strains below were used to construct the strains analyzed (Table 1) using the specified plasmids (Table 2).

**Table 1:** Strains analyzed in this study

Strain	Plasmid	Parental *E*. *coli* K-12 strain	Chromosomal editing	Clone banks
h2	pMevT	TOP10	—	m0, m1, m2, m3
h8	pMevT4	TOP10	—	m0
XL1-pMevT	pMevT	XL1	—	m0
h10	pMevT	MDS42	—	m0
h9	pMevT4	MDS42	—	m0
kle1#1	pMevT-murI	TOP10	*murI::kanR*	m1, m2, m3
h11	pMVA1	TOP10	—	m0, m1, m2, m3

**Table 2:** Plasmids used to generate strains

Plasmid	Relevant features	Reference
pMevT	p_lac_:*atoB-ERG13-tHMGR:*t_*rrnB*_, cam^R^, p15A	^30^
pMevT4	p_lac_:t_rrnB_, cam^R^, p15A	This study
pMevT-murI	p_lac_:*atoB-ERG13-tHMGR-murI:*t_*rrnB*_, cam^R^, p15A	This study
pMVA1	p_J23100_:*atoB-mvaS-mvaE:*t_*rrnB*_, cam^R^, p15A	This study
pSIM6	*exo*, *bet*, *gam*, amp^R^, pSC101^ts^	^63^

*E*. *coli* TOP10, similar to DH10B (Invitrogen):

*F− mcrA Δ(mrr-hsdRMS-mcrBC) Φ80lacZΔM15 ΔlacX74 recA1 araD139 Δ(ara*, *leu) 7697 galU galK rpsL (StrR) endA1 nupG*

*E*. *coli* XL1 (Agilent):

*recA1 endA1 gyrA96 thi-1 hsdR17 supE44 relA1 lac [F´* *proAB lacI* ^*q*^ *Z∆M15 Tn10 (Tet* ^*r*^ *)]*

*E*. *coli* MDS42 (Scarab Genomics):

MG1655 genome reduced for all ISs, *fhuACDB*, *endA* and more^46^.

Standard chemical transformation or electroporation was used for gene introduction. Strain *kle1#1* was constructed by lambda-red recombineering-based deletion of chromosomal *murI* with a kanR deletion fragment (oligos, Supplementary Table 11) in TOP10 cells harboring pSIM6 and pools of cloned RBS-variable pMevT-murI (Supplementary Table 11, construction described below). Recombineering followed standard procedures in which cells containing pSIM6 + pMevT-murI with variable RBS were grown in selectable 2xYT at 30 °C to mid-exponential phase for 3–5 h and induced at 42 °C for recombinase expression for 20 min. Subsequently, cells were spun down at 4 °C, washed in ice-cold Milli-Q H_2_O, and electroporated (1.8 kV) with the chromosomal *murI* knockout fragment, purified from PCR. Electroporated cells were then recovered in SOC medium at 37 °C for 2 h, after which cells were plated on LB agar with 50 μg/mL kanamycin + 500 μM isopropyl β-d-1-thiogalactopyranoside (IPTG) and incubated at 37 °C to continue curing of pSIM6. The selected final pMevT-murI plasmid in strain *kle1#1* contained the following *murI* RBS sequence: TCTCAC.

**Table 3:** Experimentally simulated long-term fermentations

EVO no.	Strains	Culture conditions	Clone banks	Lineages	Deep-sequenced seeds
EVO2	*h2*	2xYT, 30 °C	m0	c6–c10	All: s9 c6,c8,c10: s2-s6
EVO2B	*XL1-MevT*	2xYT, 37 °C	m0	c1–5	s6
EVO8	*h2*	2xYT + 5 g/L NaCL, 30 °C	m0	c1–4	—
	*h10*	2xYT, 30 °C	m0	c6–9	—
EVO10	*h2*	2xYT, 32 °C	m1, m2, m3	c2–4	s9
	*kle1#1*	2xYT, 32 °C	m1, m2, m3	c6–8	s9
EVO13	*h11*	2xYT, 30 °C	m0, m1, m2, m3	c1–4	s10

For test in experimentally simulated fermentations, single colonies were cultured and stored at −80 °C to serve as working clone banks (designated by *m* numbers) following standard industrial practice^61^.

### Plasmids

pMevT4 was generated by PstI digestion of pMevT followed by re-ligation of the backbone using T4 DNA ligase and standard molecular biology methods, excising the metabolic pathway cassette (*atoB*, *ERG13*, and *tHMGR*). pMevT-murI RBS variants were generated by uracil-excision cloning of *murI* into pMevT with a diversity of eight different *murI* RBS sequences added via the PCR primers for the cloning fragments (Supplementary Table 11). pMVA1 was similarly generated by uracil-excision cloning of PCR fragments, introducing a constitutive J23100 promoter with a PCR primer (Supplementary Table 11). PCRs were conducted by standard procedures with Phusion U DNA polymerase (Thermo). Uracil-excision cloning was performed by approx. equimolar mixing of the respective purified PCR products (Supplementary Table 11) in a 20 μL reaction, including 2 μL FastDigest buffer (Thermo), 0.75 μL USER enzyme (NEB), and 0.75 μL FastDigest DpnI (Thermo). The reaction incubated for 60 min at 37 °C followed by 20 min at 25 °C, and was subsequently transformed into chemically competent *E*. *coli* TOP10 cells.

### Media

For all cultivations, standard 2xYT characterization medium was used unless otherwise stated. 2xYT medium consisted of 10 g/L yeast extract (Sigma-Aldrich), 16 g/L tryptone (Bacto), 5 g/L NaCl (pH adjusted to 7.0) supplemented with 30 μg/mL chloramphenicol, and 500 μM IPTG.

For genetic transformations, SOC medium was used. SOC consisted of 5 g/L yeast extract, 20 g/L tryptone (Bacto), 10 mM NaCl, 2.5 mM KCl, 20 mM MgSO_4_, and 20 mM d-glucose.

### Simulated long-term fermentation by continuous growth

Five parallel lineages of the pMevT-harboring TOP10 clone bank *h2m0* were inoculated into aerated cultures (EVO2; Table 3). Each culture contained 25 mL medium and was grown for 8 h at 30 °C with horizontal shaking at 250 r.p.m. After 8 h, 0.5 mL broth was inoculated into 25 mL fresh medium and incubated under the same conditions for another 8 h. At each passage, the OD_600_ was recorded to determine the accumulated number of cell divisions (Supplementary Table 2). Constant pathway induction (500 μM IPTG) was applied to mimic constitutive promoter designs, as the advantages of late induction (e.g., using p_lac_) would be unattainable industrially owing to inducer cost^6^. The simulation was repeated (EVO8 and EVO13) as specified (Table 3). As backup in case of wrong experimental handling, an extra parallel lineage was often cultivated in parallel, but the analyzed lineages were always chosen randomly.

### Long-term fermentation with different *E*. *coli* host strain

Five parallel lineages of the pMevT-harboring *E*. *coli* XL1 clone bank (*XL1-MevT*) were inoculated into aerated cultures (EVO2B; Table 3). Each culture contained 50 mL medium and was grown for 12 h at 37 °C with horizontal shaking at 250 r.p.m. to match the slower growth of XL1. After 12 h, 1 mL broth was inoculated into fresh medium and incubated under the same conditions. At each passage, the OD_600_ absorbance was recorded.

### Long-term fermentation with pathway-coupled essential gene

Four parallel lineages were inoculated from, respectively, four *h2* and *kle1#1* master clone banks into aerated cultures and was cultured at 32 °C (EVO10; Table 3), but otherwise following same methods as the first simulated long-term fermentation. Randomly, three lineages of, respectively, *h2* and *kle1*#1 were selected for subsequent analysis. At each passage, the OD_600_ was recorded to determine the accumulated number of cell divisions (Supplementary Table 9). Three of four lineages were randomly selected for subsequent analysis.

### High-depth DNA sequencing and analysis

Upon each passage to new medium, 1.8 mL of the grown culture was stored at −20 °C. Similarly, 1.8 mL of grown culture was stored at the simulated fermentation end. Production plasmid populations were subsequently purified from each time point using a standard plasmid purification kit (Macherey-Nagel). The samples were then prepared for Miseq sequencing using the Nextera XT v2 set A kit (Illumina) per the manufacturer’s instructions with the addition of two “limited-cycle PCR” cycles.

Sequencing was performed in a pooled run with 150 bp paired-end reading. CLC Genomics Workbench (version 8.5) was used for initial bioinformatics analysis. First, the reads were mapped to the reference pMevT sequence (Addgene #17815). Broken aligned reads were identified using the CLC Genomics Workbench tool Breakpoint analysis to yield a table of the consensus broken unaligned reads and their abundance (maximum three mismatches allowed in the mapped read region, *p*-value for the fraction of unaligned reads set to 0.0001) to obtain an initial overview of occurred structural variation. The fraction of mobile element coverage to plasmid coverage was calculated by mapping of reads to the 24 unique *E*. *coli* DH10B mobile element sequences and the reference plasmid. Subgroups IS10R and IS10L were combined as IS10 and IS1A, B and F as IS1. Position-specific mobile element/reference coverage was calculated by mapping 80 bp sequences consisting of, respectively, the corresponding 40 bp reference plasmid and 40 bp mobile element sequence or 80 bp reference plasmid sequence. These mappings were performed with the alignment setting “global” and identity fraction set to “0.6”. Mappings without any reads covering across the mobile element junction were disregarded. SNPs and short deletions were called using the CLC Genomics Workbench Low Frequency Variant Detection tool with a 1% required significance level and 0.25% minimum frequency (unless otherwise specified). The SNP frequencies in the sequenced populations were calculated by division with their respective coverage values.

Five SNPs found in the plasmid backbone at >99% frequencies in the initial seed were regarded as present in the starting plasmid. The deep-sequencing data are available via the ArrayExpress repository (accession no: E-MTAB-5862).

### Whole-genome sequencing of single colonies

DNA for whole-genome sequencing from single colonies was extracted from a grown 2 mL culture using a standard DNA extraction kit (Qiagen), but otherwise prepared as above for a pooled Miseq run. For identification of SNPs, reads were mapped to the publicly available DH10B genome, and detected for using the Low Frequency Variant Detection tool of CLC Genomics with a minimum detection frequency of 80%.

### Measurement of mevalonic acid production by high-performance liquid chromatography

Upon each passage to a fresh culture, 900 μL medium was mixed with 900 μL 50% glycerol and stored at −80 °C. Following the simulated fermentation, each population sample from a 25 μL glycerol stock was used to inoculate 15 mL medium, and the culture was incubated at 30 °C with shaking at 250 r.p.m. for 54–58 h. Following incubation, 300 μL aliquots were treated with 23 μL 20% sulfuric acid. Samples were vigorously shaken and then spun down at 13 000 × *g* for 2 min. Supernatant (medium) samples were injected into an Ultimate 3000 high-performance liquid chromatography running a 5 mM sulfuric acid mobile phase (0.6 mL/min) on an Aminex HPX-87H ion exclusion column (300 mm × 7.8 mm, Bio-Rad Laboratories) at 50 °C. A refractive index detector was used for detection. A standard curve for mevalonic acid was generated with mevalonolactone (Sigma-Aldrich) dissolved in 2xYT medium supernatant of an engineered non-producing strain incubated under same conditions.

### Measurement of population growth rates

To measure population growth rates, 1.5 μL aliquots of stationary-phase cultures grown for productivity analysis (as described in the previous section) were used to inoculate 200 μL medium in microtiter plate wells. The microtiter plate was sealed with a Breathe-Easy polyurethane seal (USA Scientific) and was incubated at with “fast” continuous shaking in an ELx808 kinetic plate reader (BioTek), which measured the OD_630_ value every 10 min.

Background-subtracted OD_630_ values were computed using the measurements from un-inoculated wells. The local growth rates were computed for each background-subtracted OD_630_ value by regressions in rolling windows of five measurement points and background-subtracted OD_630_ values. To represent the growth rates in the actual fermentation simulations, the average was computed of the local growth rates where the background-subtracted OD_630_ was >0.04 and <0.4. R script appended (Supplementary Note 2).

### Simulation of producer fraction and model fit

The ODE system was solved analytically (Supplementary Note 1). Solution growth functions were then combined to yield a function for the fraction of producer cells in time (Supplementary Note 1). Nonlinear regression was performed to fit to this model with the nls2 R package (Supplementary Note 3). A stochastic version of the model was constructed by employing the algorithm of Gillespie to simulate discrete mutational events of our system in a stochastic manner^62^. We assume that each event (e.g., cell division and mutation) occurs according to probabilities scaled with the parameters obtained from the deterministic fit of our data (production load: 30%, escape rate: 2.1 × 10^–7^; Supplementary Note 4).

### Data availability

All relevant data are available from the authors. Deep-sequencing data from the study (Figs. 3, 4, and 6) have been deposited in ArrayExpress under ID code E-MTAB-5862 (https://www.ebi.ac.uk/arrayexpress/experiments/E-MTAB-5862).

R scripts used to process raw growth data and fit to model are provided in Supplementary Notes 2 and 3. R script for running stochastic version of model is provided in Supplementary Note 4.

## Electronic supplementary material

Supplementary Information
