# Supplementary material for: Diverse genetic error modes constrain large-scale bio-based production
Source: Nat Commun. 2018 Feb 20;9:787. doi: 10.1038/s41467-018-03232-w (PMC5820350; doi:10.1038/s41467-018-03232-w)
Supplement: Supplementary file 1 — Supplementary Information [file 41467_2018_3232_MOESM1_ESM.pdf]

Supplementary Information

Rugbjerg et al.

Diverse Genetic Error Modes Constrain Large-scale Bio-based Production

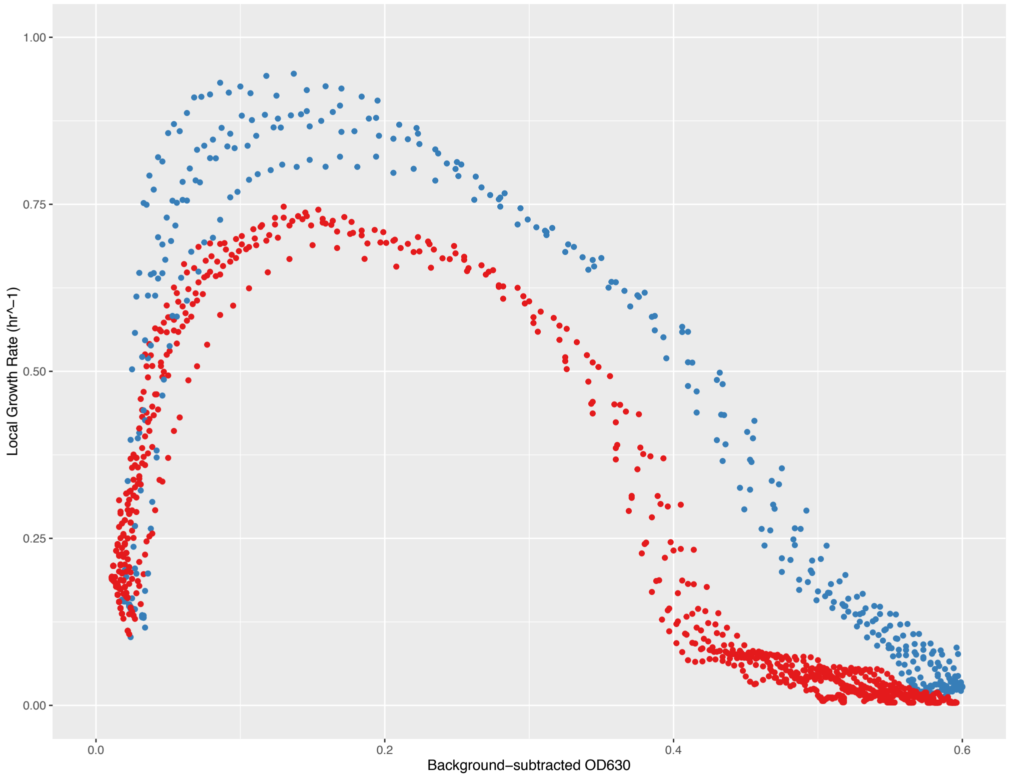

Supplementary Figure 1. Load of producing mevalonic acid (*h2m0, red*) as measured by comparison to growth of pathway-excised, non-producing control (*h8, blue*). Growth rates were dependent of the phases of growth, and therefore for quantification we use an average in the background-subtracted OD630 region 0.04-0.40, which we quantified to 30 % (n = 8) (Methods). Related to Figure 1.

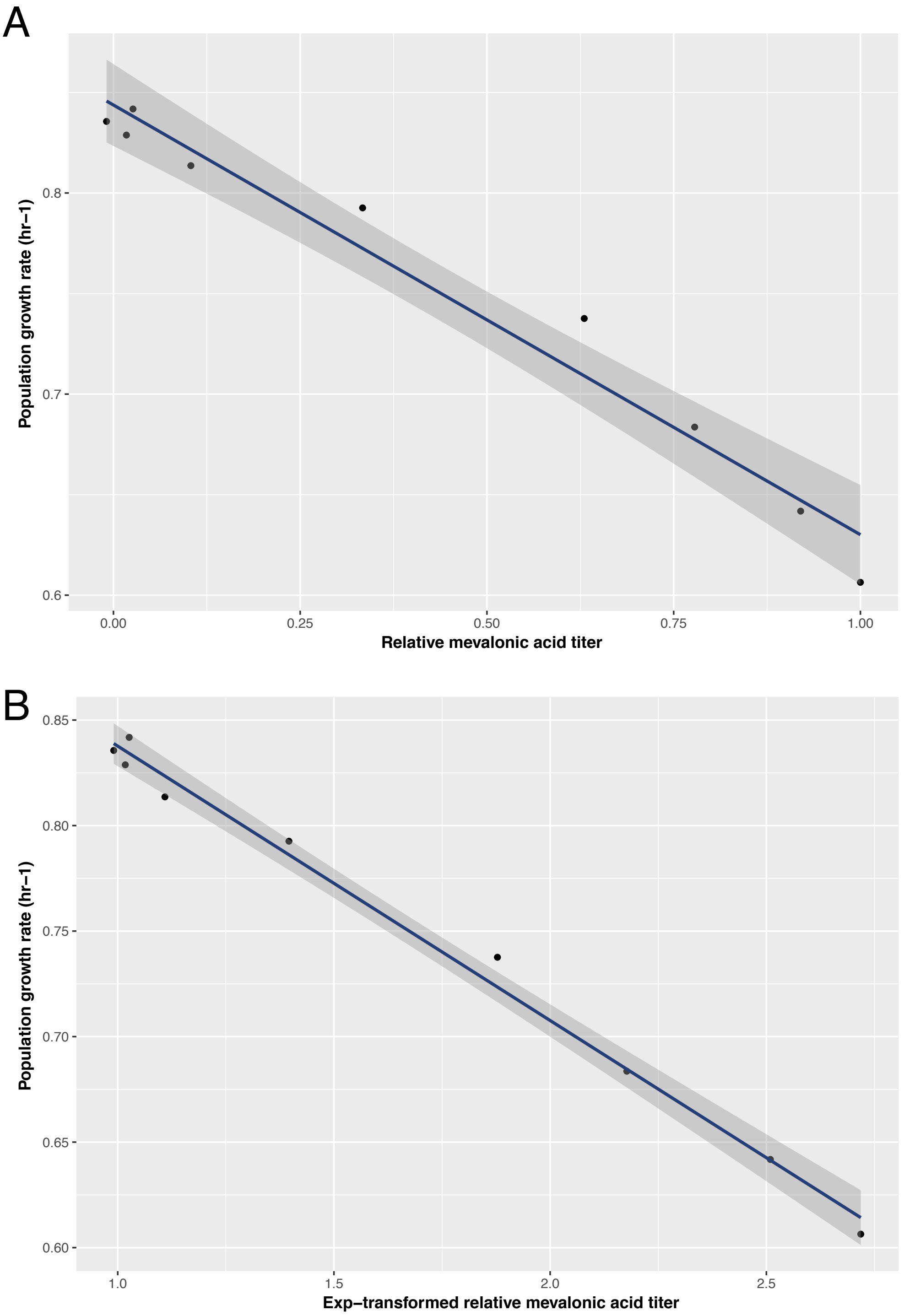

Supplementary Figure 2. The relation between population growth rate average and mevalonic acid titer of *h2m0* and long-term cultured populations (n = 5) (relative to earliest data point in simulated fermentation "EVO2". Relative mevalonic acid titer is respectively A) non-transformed, and B) log-transformed) resulting in the respective regressions shown as lines (grey area depicts 95 % confidence interval of regression): A: $y = -0.21x + 0.84$, $R^2 = 0.97$, p = 2.0 10^{-6}, and

B: $y = 0.968 - 0.13\ e^x$, $R^2 = 0.99$, p = 1.3 10^{-8}

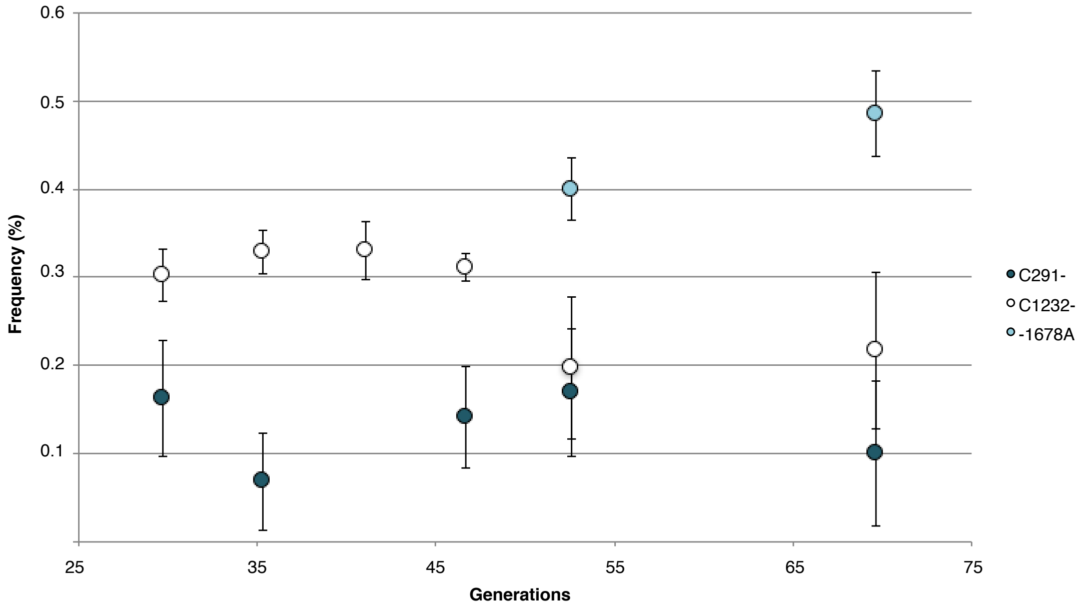

Supplementary Figure 3. Mean frequencies of three detected SNPs in the dynamically sampled lineages c6, c8 and c10 of the experimentally simulated long-term fermentation of *E. coli h2 m0,* at a minimum detection level of 0.1 %. Error bars denote standard error of the mean (n = 3).

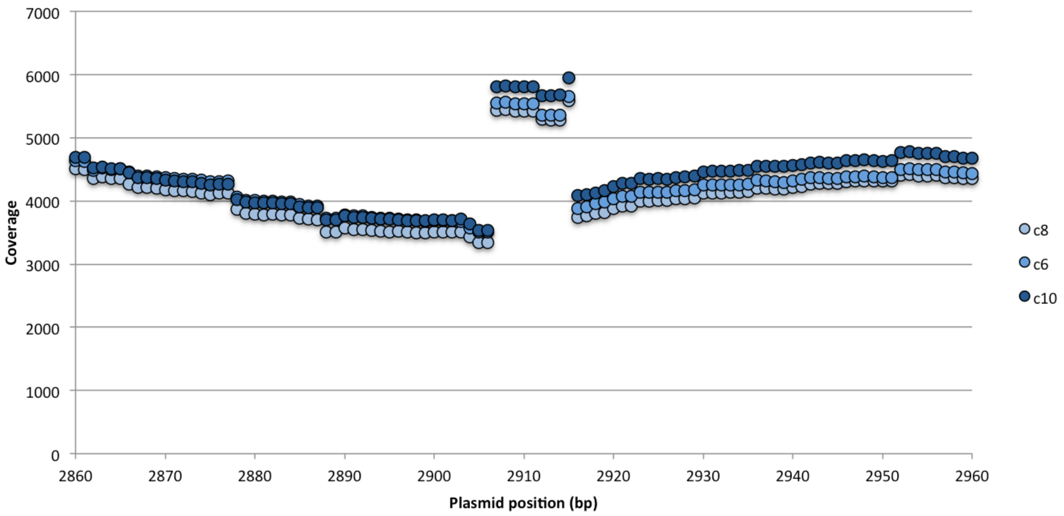

Supplementary Figure 4. Number of mapped reads (coverage) at the reference plasmid positions in the proximity of a high-frequency IS10 insertion. Jump likely results from duplication of the target recognition region (data from three parallel *h2m0* lineages c6, c8 and c10 shown following seed 9). Related to Fig. 3.

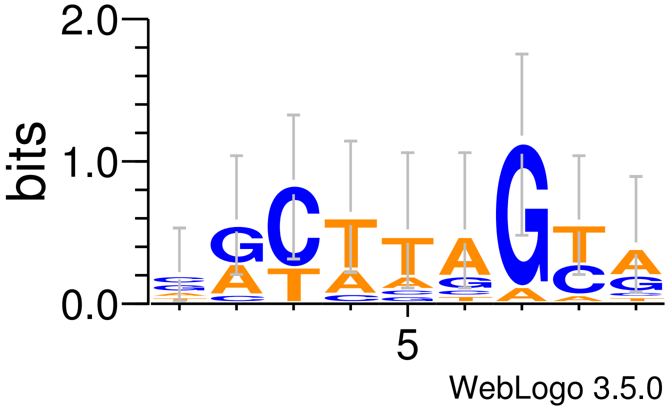

Supplementary Figure 5. Consensus sequence for ten IS10 insertion sites observed in the deep-sequenced pMevT plasmid populations. Consensus sequence analyzed using WebLogo 3 [1]. This observed consensus deviated from a previously reported consensus target sequence of IS10: NGCTNGACN [2] .

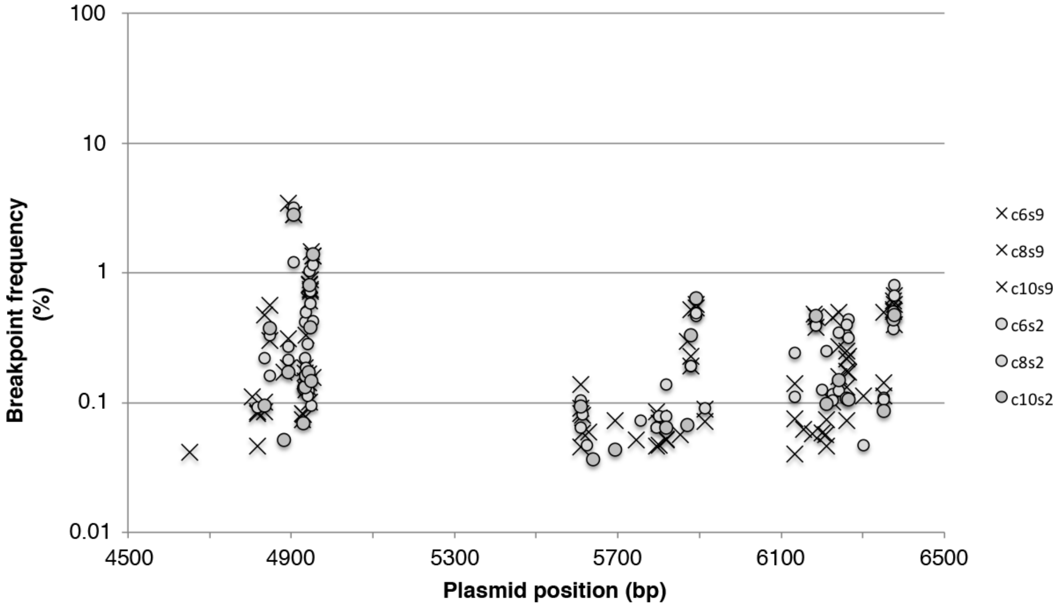

Supplementary Figure 6. Exemplary structural variation (pMevT plasmid backbone, region 4500-6500 bp featuring the *rrnB* terminator and f1 origin) without enrichment over the course of the experimentally simulated fermentation of *E. coli h2m0* “EVO2” (breakpoint frequency = breakpoint reads per coverage) lineages c6, c8 and c10, sampled after seed 2 (s2) and final seed 9 (s9).

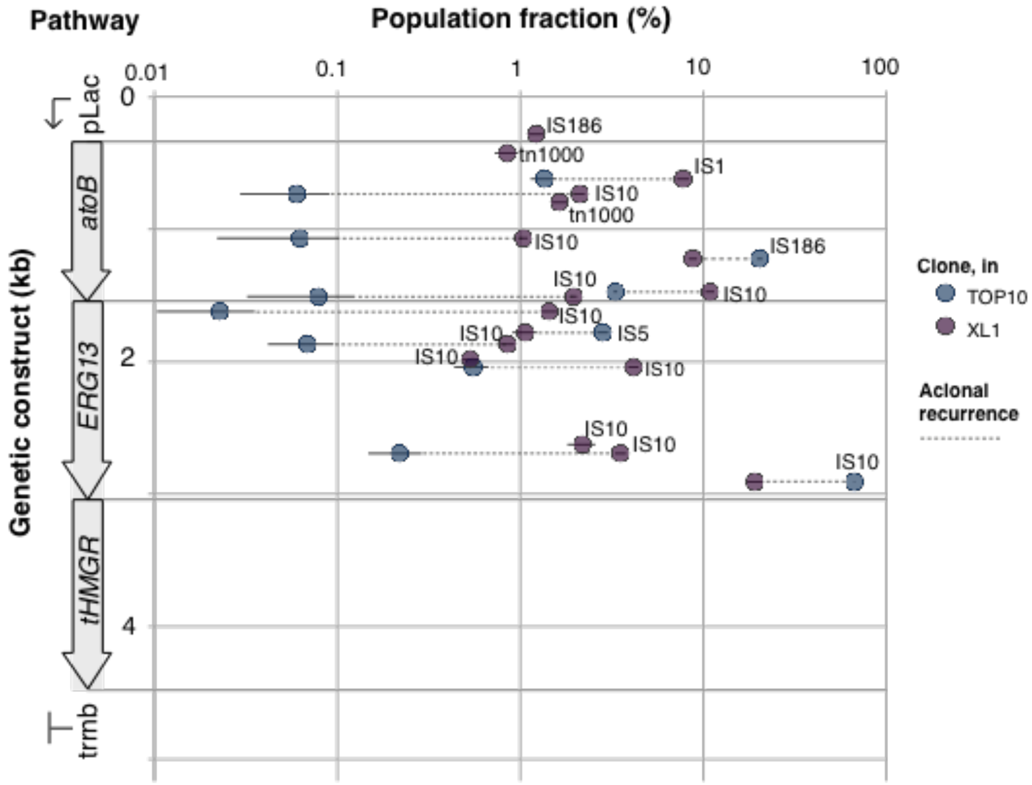

Supplementary Figure 7. Recurring mobile element disruptions within the load-carrying genetic regions of the mevalonic acid pathway in two *E. coli* production cell banks as assessed by ultra-deep sequencing of pathway populations at the end of experimentally simulated long-term fermentation ("EVO2B") with *E. coli* Top10 (*h2m0*) and XL1 (*XL1-MevTm0*) hosts. Error bars indicate standard errors (n = 5), and dashed lines connect elements recurring across the Top10 and XL1 experimentally simulated fermentations.

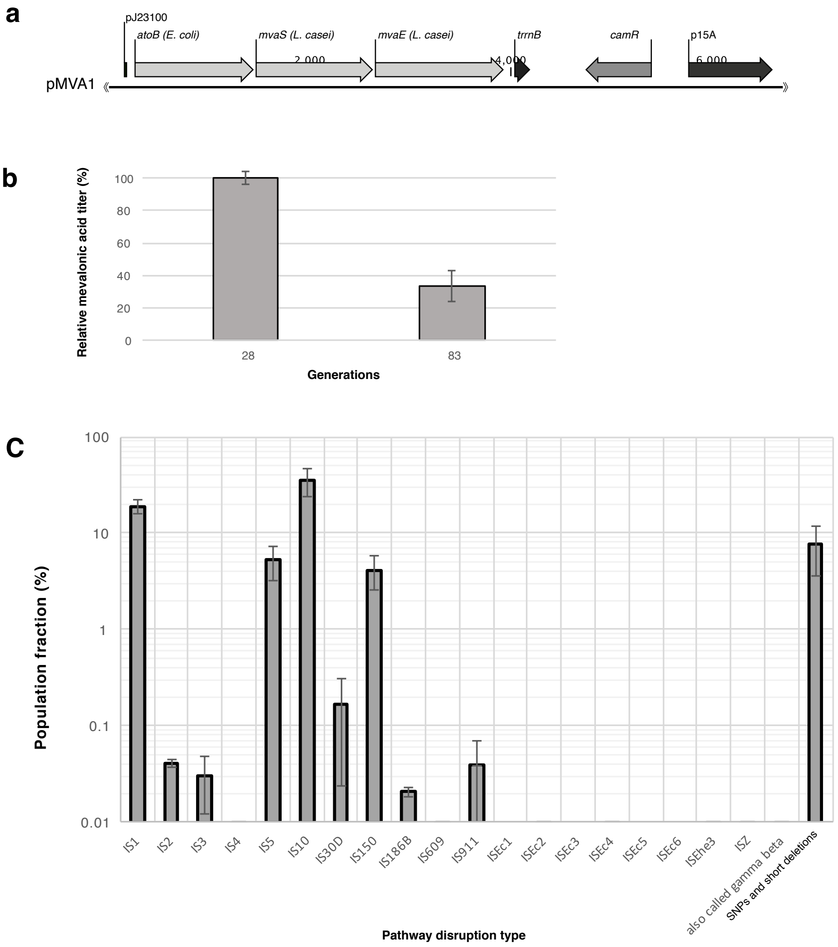

Supplementary Figure 8. Distribution of IS elements transposed into the pMVA1 plasmid populations following experimentally simulated long-term fermentation with parallel lineages of strain *h11* m0-3 for 79 generations in "EVO13", (error bars depict standard error, n = 4). SNPs and short deletions specified in Supplementary Table 8.

Supplementary Table 1. Calculated number of cell divisions (generations) needed to occupy the bioreactor type specified at the respective OD_{600} or number of cells.

Bioreactor type	Number of cells	OD_{600}	Volume (L)	Accumulated generations
Strain construction	$2\ 10^{11}$	1	0.01 L	38
Laboratory-scale	$2\ 10^{15}$	100	2 L	51
Industry-scale	$2\ 10^{17}$	100	200 L	57
Industry-scale	$2\ 10^{18}$	100	2.000 L	61
Industry-scale	$1\ 10^{19}$	100	10.000 L	63
Industry-scale	$2\ 10^{20}$	100	200.000 L	67

Supplementary Table 2. Average determined number of generations undergone by the experimentally simulated fermentations.

Growth tube (seed)	New generations (-80 stock)	Accumulated generations (-80 stock and sequenced sample)	Accumulated generations (15 mL re-grown stock for analysis)	Experimentally simulated fermentation ID and lineages	Strain clone bank
Bank culture	18.4	18.4	28.1	EVO2 c6-c10	*h2m0*
s1	5.1 +/- 0.02	23.5	33.2	EVO2 c6-c10	*h2m0*
s2	6.2 +/- 0.03	29.7	39.4	EVO2 c6-c10	*h2m0*
s3	5.6 +/- 0.04	35.3	45.0	EVO2 c6-c10	*h2m0*
s4	5.8 +/- 0.03	41.1	50.8	EVO2 c6-c10	*h2m0*
s5	5.6 +/- 0.05	46.7	56.4	EVO2 c6-c10	*h2m0*
s6	5.8 +/- 0.02	52.5	62.3	EVO2 c6-c10	*h2m0*
s7	5.7 +/- 0.00	58.2	67.9	EVO2 c6-c10	*h2m0*
s8	5.7 +/- 0.01	63.9	73.6	EVO2 c6-c10	*h2m0*
s9	5.6 +/- 0.01	69.6	79.3	EVO2 c6-c10	*h2m0*
Bank culture	18.4	18.4	28.1	EVO8 c1-4	*h2m0*
s1	9.8 +/- 0.03	28.2	37.9	EVO8 c1-4	*h2m0*
s2	5.8 +/- 0.04	38.1	47.8	EVO8 c1-4	*h2m0*
s3	5.6 +/- 0.02	43.9	53.6	EVO8 c1-4	*h2m0*
s4	5.7 +/- 0.02	49.5	59.2	EVO8 c1-4	*h2m0*
s5	5.7 +/- 0.02	55.2	64.9	EVO8 c1-4	*h2m0*
s6	5.6 +/- 0.01	60.9	70.6	EVO8 c1-4	*h2m0*
s7	5.8 +/- 0.00	66.5	76.2	EVO8 c1-4	*h2m0*
s8	5.7 +/- 0.01	72.3	82.0	EVO8 c1-4	*h2m0*
s9	5.7 +/- 0.02	77.9	87.6	EVO8 c1-4	*h2m0*
Bank culture	18.4	18.4	28.1	EVO8 c6-c9	*h10m0*
s1	9.3 +/- 0.29	27.7	37.4	EVO8 c6-c9	*h10m0*
s2	6.3 +/- 0.21	34.1	43.8	EVO8 c6-c9	*h10m0*

s3	5.8 +/- 0.07	39.9	49.6	EVO8 c6-c9	*h10m0*
s4	5.7 +/- 0.01	45.6	55.3	EVO8 c6-c9	*h10m0*
s5	5.6 +/- 0.01	51.2	60.9	EVO8 c6-c9	*h10m0*
s6	5.7 +/- 0.01	56.9	66.6	EVO8 c6-c9	*h10m0*
s7	5.6 +/- 0.00	62.5	72.2	EVO8 c6-c9	*h10m0*
s8	5.7 +/- 0.01	68.1	77.8	EVO8 c6-c9	*h10m0*
s9	5.6 +/- 0.01	73.8	83.5	EVO8 c6-c9	*h10m0*
s10	5.7 +/- 0.01	79.4	89.1	EVO8 c6-c9	*h10m0*
Bank culture	18.4	18.4	28.1	EVO13 c1-c4	*h11m0-3*
s1	10.2	28.6	38.3	EVO13 c1-c4	*h11m0-3*
s2	5.4	34.0	43.7	EVO13 c1-c4	*h11m0-3*
s3	5.7	39.7	49.4	EVO13 c1-c4	*h11m0-3*
s4	6.1	45.8	55.5	EVO13 c1-c4	*h11m0-3*
s5	5.2	51.0	60.7	EVO13 c1-c4	*h11m0-3*
s6	5.7	56.7	66.4	EVO13 c1-c4	*h11m0-3*
s7	5.7	62.4	72.1	EVO13 c1-c4	*h11m0-3*
s8	5.6	68.0	77.7	EVO13 c1-c4	*h11m0-3*
s9	5.6	73.6	83.3	EVO13 c1-c4	*h11m0-3*
s10	5.7	79.4	89.1	EVO13 c1-c4	*h11m0-3*

Long-term fermentations studied the mevalonic acid-producing *h2m0, h10m0* and *h11m0-3* clone banks (in EVO2 and EVO8), accumulated and at the individual 8-hour passages (seeds) (standard error shown +/-, for EVO2: n = 5, EVO8: n = 4, EVO13: n = 4).

Supplementary Table 3. Measured mevalonic acid titers (g· L^{-1}) in earliest time point from the indicated simulated fermentation.

**Strain clone bank**	**Simulated fermentation ID**	**Cultivation condition**	**Mevalonic acid ($g \cdot L^{-1}$) +/- se**
h2 m0	EVO2	Std. medium, 30 deg. C	1.2 +/- 0.04
h2 m0	EVO8	Opt. medium, 30 deg. C	0.9 +/- 0.04
h10 m0	EVO8	Std. medium, 30 deg. C	0.5 +/- 0.05
h2 m1, m2, m3	EVO10	Std. medium, 32 deg. C	1.3 +/- 0.04
kle1#1 m1, m2, m3	EVO10	Std. medium, 32 deg. C	1.0 +/- 0.04
h11 m0, m2, m3, m4	EVO13	Std. medium, 30 deg. C	1.3 +/- 0.05

se: standard error of the mean (EVO2: n = 5, EVO8: n = 4, EVO10: n = 3, EVO13: n = 4). Related to Figure 1.

Supplementary Table 4. Variation in the cell divisions (generations) till first mutation by four replicate runs of a stochastic version of our population escape model (script in Supplementary Note 3).

Replicate	Generations to first mutation	No. of cells
1	19.42837	705542
2	19.44143	711956
3	20.23096	1230621
4	20.99365	2087940

Simulations performed on an 8-core desktop computer (Intel® Core™ i7-4770K CPU @ 3.50GHz × 8). Computation time scales with number of cells and ran >3 days to observe a first mutation (escape) in all four replicates.

Supplementary Table 5. Production loads and escape rates estimated from fermentation simulations by fit to model or by pure-culture measurements.

**Parameter**	***Strain* and condition**	**Method**	**Data source**	**Estimate value (generation^{-1}) and p-value**
Production load	*h2m0*, relative to *h8* std. medium	Growth rate average	Pure-culture growth curves	30 %
Escape rate	*h2m0*, std. medium	Fit to model using pure-culture determined production load	Production over time	$2.5 \cdot 10^{-8}$ (p-value = 0.0012)
Escape rate	*h2m0*, std. medium	Fit to model using pure-culture determined production load	Total IS fraction over time (from deep-seq)	$8.7 \cdot 10^{-8}$ (p-value < 0.0001)
Production load	*h2m0*, std. medium	Free fit to model	Total mobile element fraction over time (from deep-seq)	28 % (p-value < 0.0001)
Escape rate	*h2m0*, std. medium	Free fit to model	Total mobile element fraction over time (from deep-seq)	$2.1 \cdot 10^{-7}$ (p-value < 0.0001)
Production load	*h2m0*, relative to *h8* opt. medium	Growth rate average	Pure-culture growth curves	23 %
Production load	*h10m0*, relative to *h9* std. medium	Growth rate average	Pure-culture growth curves	26 %

Production load	*h2m0*,<br>std. medium	Fit to model with NGS-determined escape rate	Production over time	26 %<br>($p < 0.0001$)
Production load	*h2m0*,<br>opt. medium	Fit to model with NGS-determined escape rate	Production over time	21 %<br>($p < 0.0001$)

Supplementary Table 6. SNPs found in the chromosomes of colonies picked from streaks from the end-point experimentally simulated fermentations of *h2m0* (std. medium, “EVO2”).

Sample colony	Region	Type	Reference	Allele	Coverage	Frequency	Probability
TOP10	4272971	Insertion	-	T	36	97.22	1
c6s9k1	4272971	Insertion	-	T	25	100	1
c6s9k2	4272971	Insertion	-	T	24	100	1
c6s9k3	4272971	Insertion	-	T	46	97.83	1
c8s9k1	4272971	Insertion	-	T	35	97.14	1
c8s9k2	4272971	Insertion	-	T	40	95	1
c8s9k3	4272971	Insertion	-	T	42	100	1
c10s9k1	4272971	Insertion	-	T	25	100	1
c10s9k2	4272971	Insertion	-	T	24	100	1
c10s9k3	4272971	Insertion	-	T	46	97.83	1

SNPs were identified after mapping of reads to the publicly available genome sequence of *E. coli* DH10B (accession CP000948). SNPs were called at minimum coverage of 15, maximum coverage of 1000. Three colonies (k1-3) were randomly picked from lineage c6, c8 and c10 respectively.

Supplementary Table 7. Summary statistics of linear regressions of log-transformed mobile element subgroup fractions with cell divisions.

IS subgroup	Estimate	Std. Error	t value	Pr(>|t|)	R^2
IS10	0.103524	0.006623	15.63	0.000569	0.98
IS186	0.099346	0.004302	23.09	0.000178	0.99
IS5	0.052057	0.006731	7,734	0.0045	0.95
IS1	0.05529	0.007353	7,519	0.004875	0.95
IS3	0.066145	0.004971	13.31	0.000917	0.98
IS150	0.015975	0.003622	4.41	0.021625	0.86
tn1000	0.083741	0.003768	22.23	0.000199	0.99
IS2	0.022302	0.005604	3.98	0.028387	0.84
IS911	0.01759	0.00377	4,667	0.0186	0.88
IS30D	0.03692	0.01234	2,993	0.05799	0.75

Enrichment rates were calculated by linear regression of log10-transformed mobile element frequencies in the exponential phase seeds s2-s6 for the three time-lapse sequenced samples lineages c6, c8 and c10. Related to Fig. 4.

Supplementary Table 8. SNPs and short deletions in the pMVA1 plasmid populations in the four end-point deep-sequenced lineages m0-3 of *E. coli h11*.

Lineage	Plasmid region	Annotation	Type	Reference sequence	Variant sequence	Frequency (%)
m0	165..173	p_{J23100}	Deletion	CTAGGTACA	-	20.24
m0	466	*atoB*	SNV	T	A	0.33
m0	1107	*atoB*	Deletion	C	-	0.33
m0	240..249	5'-UTR *atoB*	Deletion	GAGGAGAAAG	-	0.29
m1	1383	*atoB*	SNV	A	G	0.32
m1	1941	*mvaS*	SNV	T	G	0.27
m1	1107	*atoB*	Deletion	C	-	0.24
m2	341	*atoB*	Deletion	G	-	6.35
m2	1107	*atoB*	Deletion	C	-	0.36
m2	164..173	p_{J23100}	Deletion	CCTAGGTACA	-	0.20
m3	461	*atoB*	SNV	A	G	0.58
m3	459^460	*atoB*	Insertion	-	C	0.52
m3	1107	*atoB*	Deletion	C	-	0.26
m3	1145	*atoB*	SNV	G	C	0.23

SNPs called at a minimum detection frequency of 0.2 %.

Supplementary Table 9. Average number of generations undergone by the experimentally simulated fermentation of the mevalonic acid-producing *h2* (m1-m3) and *kle1#1* (m1-m3) (EVO10).

**Growth tube (seed)**	**New generations (-80 stock)**	**Accumulated generations (-80 stock)**	**Accumulated generations (15 mL re-grown stock for analysis)**	**Strain and cell banks**
Bank culture	18.4	18.4	28.1	h2 m1-m3
s1	6.2 +/- 0.06	24.5	34.3	h2 m1-m3
s2	5.4 +/- 0.03	30.0	39.6	h2 m1-m3
s3	5.9 +/- 0.01	35.7	45.5	h2 m1-m3
s4	5.5 +/- 0.02	41.2	51.0	h2 m1-m3
s5	5.8 +/- 0.03	46.9	56.8	h2 m1-m3
s6	5.9 +/- 0.03	52.7	62.7	h2 m1-m3
s7	5.8 +/- 0.01	58.4	68.4	h2 m1-m3
s8	5.6 +/- 0.02	64.3	74.0	h2 m1-m3
s9	5.6 +/- 0.00	70.0	79.6	h2 m1-m3
Bank culture	18.4	18.4	28.1	kle1#1 m1-m3
s1	6.1 +/- 0.04	24.5	34.2	kle1#1 m1-m3
s2	5.5 +/- 0.01	30.0	39.7	kle1#1 m1-m3
s3	5.7 +/- 0.02	35.7	45.4	kle1#1 m1-m3
s4	5.6 +/- 0.02	41.2	50.9	kle1#1 m1-m3
s5	5.7 +/- 0.00	46.9	56.6	kle1#1 m1-m3
s6	5.7 +/- 0.03	52.7	62.4	kle1#1 m1-m3
s7	5.7 +/- 0.02	58.4	68.1	kle1#1 m1-m3
s8	5.9 +/- 0.01	64.3	74.0	kle1#1 m1-m3
s9	5.7 +/- 0.01	70.0	79.7	kle1#1 m1-m3

Generations shown as accumulated and at the individual 8-hour passages (seeds) (standard error shown +/-, n = 3). Related to Fig 6.

Supplementary Table 10. Population frequencies (%) of SNPs detected in lineages from of experimentally simulated fermentations with mevalonic acid-producing *E. coli* ("EVO10").

**Strain (expression plasmid)**	**Cell bank**	**C1232- (*atoB*) (%)**	**-1678A (*ERG13*) (%)**	**A1678- (*ERG13*) (%)**
h2 (pMevT)	m1	0.37	0.26	<0.25
	m2	0.27	1.15	<0.25
	m3	0.36	0.42	<0.25
kle1#1 (pMevT-murI1)	m1	0.30	1.95	<0.25
	m2	0.37	<0.25	15.22
	m3	<0.25	<0.25	<0.25

Related to Fig. 6. Positions are indicated relative to the sequence of the pMevT plasmid sequence.

Supplementary Table 11. Oligo sequences used for assembly of indicated plasmids and *E. coli murI* deletion fragment.

Oligo ID	DNA sequence (5'-)	N in RBS (in oligo)	Predicted log10-relative RBS strength [3]	DNA template for PCR	Plasmid assembled from this PCR
P642	ACAAATAAGUCGACCTGCAGGCATGCAAG	-	-	pMevT	pMevT-murI RBS variants
P649	ATACCTUNTCACACCTTAGGATTTAATGCAGGTGACGG	A	0.197		
		C	0.222		
		T	0.205		
		G	0.179		
P642	ACAAATAAGUCGACCTGCAGGCATGCAAG	-	-	pMevT	pMevT-murI RBS variants
P661	ATACCTUNTGAGACCTTAGGATTTAATGCAGGTGACGG	A	0.184		
		C	0.178		
		T	0.186		
		**G**	0.186		
P650	AAGGTAUGGCTACCAAACTGCAGGACGGGA	-	-	*E. coli* XL1 gDNA (murI insert)	pMevT-murI RBS variants
P651	ACTTATTTGUTCAGCCTAAAACTGCCAGTTTTTCGAGCG	-	-		
P654	CAGGACGGGAATACACCTTGTCTGGCAGCTACAC CTTCTGAACCACGTCCGTGTAGGCTGGAGCTGCTTC	-	-	*E. coli gDNA harboring the Keio collection kanR deletion cassette* [4]	-
P655	TGACCGCGCAACATTCAACCAAATCAGCCTAAAA CTGCCAGTTTTTCGAGATTCCGGGGATCCGTCGACC	-	-		-
P423	ATCCTGACGGUACCGCTACCAGCGGTGGTTTG	-	-	pMevT[5]	pMVA1
P452	AATGAGUCGCTTTCCAGTCGGGAAA	-	-		
P451	ACTCATUgacggctagctcagtcctaggtacagt gctagcATTACGCCAAGCGCGCAATT	-	-	pMevT[5]	pMVA1
P517	aattcAGCUTTTGTTCCCTTTAGTGAG	-	-		
P516	AGTCGACCUGCAGGCATG	-	-	pMevT[5]	pMVA1
P535	ACCGTCAGGAUGGCCTTCTGCTTAATTTGATGCCT	-	-		
P514	AGCTgaatUcattaaagaggagaaaggtacc	-	-	pMEV7[6]	pMVA1
P515	AGGTCGACUCAATCCCGATTTTCATCTTT	-	-		

Supplementary Note 1

A simple mathematical model for the fraction of producers in time is established by solution of an ordinary differential equation system (eq1 and eq2) with analogy to models of plasmid loss dynamics[7].

p(t): producer cells in time

np(t): non-producer cells in time

μ: specific growth rate

ρ: production load = $1 - \frac{\mu_p}{\mu_{\mathrm{np}}}$

(eq1): $$\frac{dp(t)}{dt} = \mu_p \cdot p(t) - k_{\mathrm{escape}} \cdot p(t)$$

(eq2): $$\frac{d\mathrm{np}(t)}{dt} = \mu_{\mathrm{np}} \cdot \mathrm{np}(t) + k_{\mathrm{escape}} \cdot p(t)$$

Non-producer cells are converted from producer cells at a rate k_{escape} and such non-producers alleviate a reduction in growth rate resulting from the production load ρ. The solution growth functions (eq3 and eq4) assume a pure initial inoculum of a single, producing cell, and can be found by first solving eq1 and inserting the resulting function $p(t)$ in eq2.

(eq3): $$p(t) = \mathrm{e}^{(\mu_p - k_{\mathrm{escape}})\cdot t}$$

(eq4): $$\mathrm{np}(t) = k_{\mathrm{escape}} \frac{1-\mathrm{e}^{(\mu_p - \mu_{\mathrm{np}} - k_{\mathrm{escape}})t}}{k_{\mathrm{escape}} + \mu_{\mathrm{np}} - \mu_p} \mathrm{e}^{\mu_{np}\cdot t} = k_{\mathrm{escape}} \frac{1\text{-}\mathrm{e}^{\text{-}(\rho\cdot\mu_{\mathrm{np}} + k_{\mathrm{escape}})t}}{k_{\mathrm{escape}} + \rho\cdot\mu_{\mathrm{np}}} \mathrm{e}^{\mu_{\mathrm{np}}\cdot t}$$

From these, the fraction of producers in time (eq5) can be derived.

(eq5): $$\frac{p(t)}{p(t)+\mathrm{np}(t)} = \frac{\mathrm{e}^{(\mu_p - k_{\mathrm{escape}})\cdot t}}{k_{\mathrm{escape}} \frac{1-\mathrm{e}^{-(\rho\cdot\mu_{\mathrm{np}} + k_{\mathrm{escape}})t}}{k_{\mathrm{escape}} + \rho\cdot\mu_{\mathrm{np}}} \mathrm{e}^{\mu_{\mathrm{np}}\cdot t} + \mathrm{e}^{(\mu_p - k_{\mathrm{escape}})\cdot t}} = \frac{k_{\mathrm{escape}} + \rho\cdot\mu_{\mathrm{np}}}{k_{\mathrm{escape}}\cdot \mathrm{e}^{(k_{\mathrm{escape}} + \rho\cdot\mu_{\mathrm{np}})\cdot t} + \rho\cdot\mu_{\mathrm{np}}}$$

By defining μ_{p} relative to $\mu_{\mathrm{np}} = 1$, the model is only dependent on the relative growth rate (ρ) and the escape rate (k_{escape}).

Supplementary Note 2

```
#R Script to analyze growth rate measurement

#Required packages

library(ggplot2)

library(reshape2)

library(scales)

library(plyr)

library(drc)

library(zoo)

library(stringr)



#Define TimeConverter function

TimeConverter <- function(z){

  cTime <- as.character(z[,1])

  Time <- (sapply(strsplit(cTime,":"),

                  function(x) {

                    x <- as.numeric(x)

                    round(x[1]*60+x[2]+x[3]/60, digits = 0)

                  }

  )

  )

  return(Time)

}

#Mac

data <- na.omit(read.delim("~/s1-s9_Data.csv", header=T, skip =0, sep = ";"))

map <- read.csv("~/Growth_map_EVO2_populations.csv",sep=";", header = T, colClasses =
c(rep("character", 2), "numeric"))

#Convert time to min

names(data)[1] = "Time" #Standardize naming

data$Time <- TimeConverter(data)

#AP: Melt the data if not in long format already

x.data <- melt(data, id = "Time")



#AP: Match values from mapping file to data

x.data$Strain<-map[match(x.data$variable, map$Well),2]

x.data$Replicate<-as.factor(map[match(x.data$variable, map$Well),3])

names(x.data)<-c("Time","Well","OD","Strain","Replicate")
```

```
x.data$OD<-as.numeric(x.data$OD)

#Set bgOD:
avg.bkg = 0.115
#Subtract background
x.data$bkgOD <- x.data$OD - avg.bkg
#PR: Add ln data
x.data$lnOD <- log(x.data$bkgOD)
#Add time in hours
x.data$Timehr <- x.data$Time/60


#PR: First subset x.data to exclude the BKG wells
x2.data <- subset(x.data, lnOD != "NA" & Strain != "BKG")
x2.data$lnOD <- as.numeric(str_replace_all(x2.data$lnOD, "Inf", "0"))
x2.data$lnOD <- as.numeric(str_replace_all(x2.data$lnOD, "NA", "0"))
x2.data$lnOD <- as.numeric(str_replace_all(x2.data$lnOD, "NaN", "0"))

#AP: Define the window size of rolling regression
WinSize <- 5
#AP: Define time and OD values for regression:
x <- as.numeric(x2.data$Timehr) #E.g. hours or minutes.
y <- as.numeric(x2.data$lnOD)
z <- as.data.frame(cbind(x,y))

#Standardize naming:
names(z)<-c("Time","lnOD")

#Fit data with a
RollFit<-rollapply(zoo(z), width=WinSize,
function(Z) {
reg<-lm(formula=Time~lnOD, data = as.data.frame(Z)) #Apply linear regression to lnOD against time.
cbind(1/coef(reg)[2],summary(reg)$r.squared)}, #calculate 1/slope
by.column=FALSE, align="right")

#Standardize naming:
names(RollFit)<-c("LocalGrowthRate","Rsquared")

#Attach to existing dataframe *NB: Fit values start at the row corresponding to WinSize!
x3.data <- cbind(x2.data[WinSize:nrow(x2.data),],as.data.frame(RollFit))

xr.data<-subset(x3.data)
```

Supplementary Note 3

R script

```
library(nls2)

#Fraction of producing cells at time 0 (set equal to 1 to follow the model described

#in the manuscript)

Fpc0<-1


#Load total mobile element fractions (NGS-determined)

ISobs <- data.frame(c(29.7,35.3,41.1,46.7,52.5,69.9))

colnames(ISobs)[1] <- "generations"

ISobs$fraction <-

c(0.991197834,0.980170492,0.919041917,0.733211699,0.338929329,0.000657726)

z <- nls2(fraction ~ ((u+ro)*Fpc0)/((u+ro*(1-Fpc0))*exp((u+ro)*generations)+ro*Fpc0),

data = ISobs,

          start = list(u=0.005,ro=0.1), control = list(maxiter = 5000))

summary(z)
```

Supplementary Note 4

R script

```
#Load required libraries

library(ggplot2)

library(foreach)

library(doParallel)


#setup parallel backend to use multiple processors

cl<-makeCluster(4)

registerDoParallel(cl)



###set parameters for growth of producers (gP), non-producers (pNP) and escape rate
(k)###

parms=c(gP=0.72,gNP=1,k=2.1E-7)

initial=c(P=29, NP=1)

time.window=c(0, 15)


# define how state variables for cell division and mutational escape

processes <- matrix(0, nrow=3, ncol=2,

                    dimnames=list(c("birth P",

                                    "Escape P",

                                    "birth NP"),

                                  c("P","NP")))


processes[1,1]=1 #If birth of P

processes[2,1]=-1 #If escape of P

processes[2,2]= 1

processes[3,2]=1 #If birth of NP


# process probabilities

probabilities <- function(state){

  P<-state[1] #Define parameters

  NP<-state[2]

  gP<-parms[1]; gNP<-parms[2];k<-parms[3]
```

```
  a1<-gP*P #P birth
  a2<-k*P #Escape
  a3<-gNP*NP #NP birth

  a<-c(a1,a2,a3)
  names(a)<-c("a1","a2","a3")
  a
}



### Initiate parallelized loop: ###
ls <- foreach(n = 1:4) %dopar% {

  # initialize state and time variables and write them into output table
  state <- initial
  time <-  time.window[1]

  # define output dataframe
  output <- data.frame(t=time,
                       P=state["P"], NP=state["NP"],
                       row.names=1)


  #start timer to get time of  simulation
  strt<-as.numeric(Sys.time())

 #Define stop-conditions for each simulation e.g. fraction of producers or absulute
time (in seconds)
  while(state["P"]/(state["P"]+state["NP"])>0.5 & as.numeric((Sys.time()-
wst))<252000){

    #calculate process probabilities for current state
    a<-probabilities(state)

    #WHEN does the next process happen?
    tau<-rexp(1,rate=sum(a))
```

```
    #update time
    time = time+tau

    #WHICH process happens after tau?
    act<-sample(length(a), 1, prob=a)

    #Update states
    state<-processes[act,]+state

    #write into output
    output <- rbind(output,c(time,state))
  }

  #Add info on e.g. replicate and parameters
  output$ER <- "2.1*10^-7" #Escape rate
  output$rep <- n #Replicate
  output$frac <- output$P/(output$P+output$NP) #Producer fraction

  #Write each simulation to a file (optional)
  write.table(output, file=file.path("File path", paste("Filename",".txt", sep="")),
              sep="\t", row.names=F)

  output

}

#Show simulation time
as.numeric((Sys.time()-wst))/60/60

#End parallelization
stopCluster(cl)

#Combine output to a dataframe
SDF<-as.data.frame(ls[[1]])

for( i in 2:length(ls)) {
  SDF <- rbind(SDF,as.data.frame(ls[[i]]))
}
```

```
#Calculate generations
SDF$Gen <- log10(SDF$P+SDF$NP)/log10(2)

#Plot output

ggplot(SDF, aes(x = Gen, y = frac, color = as.factor(rep)))+
  geom_line(size=1)+
  ylab("Producer fraction")+
  xlab("Generations")+
  ggtitle("Stoch. simulation")
```

Supplementary References

1. Crooks, G., Hon, G., Chandonia, J. & Brenner, S. WebLogo: a sequence logo generator. *Genome Res.* **14,** 1188–1190 (2004).
2. Craig, N. L. Target site selection in transposition. *Annu. Rev. Biochem.* **66,** 437–474 (1997).
3. Bonde, M. T. *et al.* Predictable tuning of protein expression in bacteria. *Nat. Methods* **13,** (2016).
4. Baba, T. *et al.* Construction of Escherichia coli K-12 in-frame, single-gene knockout mutants: the Keio collection. *Mol. Syst. Biol.* **2,** 2006.0008 (2006).
5. Martin, V. J. J., Pitera, D. J., Withers, S. T., Newman, J. D. & Keasling, J. D. Engineering a mevalonate pathway in Escherichia coli for production of terpenoids. *Nat. Biotechnol.* **21,** 796–802 (2003).
6. Xiong, M., Schneiderman, D. K., Bates, F. S., Hillmyer, M. a & Zhang, K. Scalable production of mechanically tunable block polymers from sugar. *Proc. Natl. Acad. Sci. U. S. A.* **111,** 8357–62 (2014).
7. Proctor, G. N. Mathematics of microbial plasmid instability and subsequent differential growth of plasmid-free and plasmid-containing cells, relevant to the analysis of experimental colony number data. *Plasmid* **32,** 101–130 (1994).
